# Infant rat ultrasonic vocalizations in the neurodevelopmental model of schizophrenia

**DOI:** 10.1038/s41598-025-08412-5

**Published:** 2025-07-28

**Authors:** Agnieszka Potasiewicz, Zuzanna Mincikiewicz, Piotr Popik, Agnieszka Nikiforuk

**Affiliations:** Department of Behavioral Neuroscience and Drug Development, Maj Institute of Pharmacology, Polish Academy of Sciences, 12 Smetna Street, 31-343 Kraków, Poland

**Keywords:** Ultrasonic communication, Neonatal vocalization impairments, Unsupervised clustering, Syntactic deficits, Vocal sequencing, Social behaviour, Emotion, Cognitive neuroscience

## Abstract

Schizophrenia is characterized by early brain developmental abnormalities resulting in, among others, compromised communication. Rodent models, such as prenatal exposure to methylazoxymethanol acetate (MAM), help investigate schizophrenia-related deficits. Ultrasonic vocalizations (USVs) in rodent pups are a preclinical tool to study social communication. This study examines how prenatal MAM exposure affects the development, structure, and maternal potentiation of USVs. Pregnant rats received MAM or saline on the 17th gestational day. Offspring USVs were recorded upon maternal separation on the 6th, 9th, and 12th postnatal days (PND), while maternal potentiation was tested on the 10th PND. USV characteristics, temporal organization, clustering, and syntax were analyzed by DeepSqueak’s machine-learning algorithms. Unlike controls, which showed an increasing USV rate associated with proper vocal development, MAM-exposed pups displayed a stable emission rate across days and emitted fewer USVs on the 12th PND. Maternal potentiation was weak or absent in MAM pups, which also exhibited lower call complexity (reduced bandwidth and frequency) and longer duration. Temporal analysis revealed delayed vocal onset, prolonged inter-call intervals, and disrupted call arrangement. Syntax analysis indicated a simplified transition pattern dominated by low-pitched flat calls. Taken together, prenatal MAM exposure disrupts vocal communication, leading to less complex vocalizations with altered timing and structure. These deficits may serve as early markers of negative-like symptoms or cognitive dysfunctions in schizophrenia.

## Introduction

The negative symptoms of schizophrenia include impaired verbal communication^1,2^. Unlike “positive” and cognitive symptoms, the negative manifestations of the disorder—such as low motivation, apathy, anhedonia, reduced emotional expression, and limited speech—are more persistent, difficult to heal, and greatly impact daily life and patients’ well-being^3–5^.

Schizophrenia, typically diagnosed in early adulthood, often stems from early brain development abnormalities, leading to subtle deficits in communication, sociability, motor skills, emotional expression, and cognitive functioning that emerge from infancy through adolescence^6,7^. These early signs are critical for predicting disease outcomes and represent a key window for preventive interventions^8^. Notably, recent advancements in automated speech analysis have shown high accuracy in identifying children at risk of psychosis, often surpassing traditional clinical assessments^9–12^.

Schizophrenia-related communication deficits appear early in life and manifest in reduced verbal productivity (shorter sentences, limited vocabulary, simpler syntax) and decreased fluency (delayed responses, long pauses, sequencing issues)^9,13–20^.

Communication and sequencing abnormalities tend to worsen over time and are strong predictors of poor outcomes^16^. Preclinical studies can investigate these aspects by analyzing rats’ ultrasonic vocalizations (USVs). USVs are essential for communication between individuals and serve as indicators of the rats’ emotional states^21^. Rodent auditory communication primarily occurs at ultrasonic frequencies beyond the range of human hearing^21^. Rat infant USVs are an early form of communication between pups and their mothers. During the first three weeks of life, when they depend entirely on parental care, pups emit calls within the range of 40–130 kHz (so-called “40 kHz USVs”) to communicate with mothers and increase their chances of survival. Like the functions of babies’ cries^22,23^, isolation-induced USVs trigger search-and-retrieval behaviors in dams^24^. For instance, playback of pup USVs causes similar retrieval behaviors in dams as those caused by isolated vocalizing pups^25^. Pup USVs can be generally classified into short, flat, and frequency-modulated subtypes^26^. The temporal organization of pup vocalizations, such as call rate and inter-call interval, is essential for eliciting maternal care^25,27–29^. Additionally, the syntactic structure of USVs is critical for communication, as subtypes follow a specific syllabic arrangement in phrases and motifs^25,29–31^.

The developmental curve of neonatal USVs is linked to growing independence from caregivers and is strongly safeguarded by intrinsic mechanisms^32^. A key aspect of the distress call is the maternal potentiation effect, where pups increase their vocalization rate during separation that follows a brief contact with the mother. Maternal potentiation is regarded as a model for studying early social bonds, attachment, and the neurobiological mechanisms underlying social communication and maternal care. USVs also serve as a valuable tool for examining how genetic, environmental, and neurochemical factors shape the development of social behavior and emotional regulation during early life^22,33–38^.

USVs have been recognized as a preclinical tool for modeling aspects of neuropsychiatric diseases^39^. In rodents, the postnatal days (PND) 6th–12th correspond developmentally to late gestation through infancy in humans, supporting the translational relevance of this period for modeling early risk mechanisms associated with neurodevelopmental disorders^40^. While rodent USVs are well-studied in autism models^41–51^, research on schizophrenia-like impairments remains limited, with few studies examining call characteristics and even fewer exploring the temporal and sequential structure of USV bouts, especially in the early developmental stage^26,47,52–54^.

Methylazoxymethanol acetate (MAM) is a neurotoxin and a DNA methylating agent that disrupts neurogenesis when administered during critical periods of fetal brain development. Exposure to the MAM on 17th embryonic day leads to behavioral (e.g., hyperactivity, social deficits, impaired sensorimotor gating and cognition), anatomical (e.g., reduced cortical volume), and neurophysiological (e.g., disrupted corticocortical transmission) abnormalities in adult offspring that mirror core features of schizophrenia^23,50,51^. The effects of MAM can be detected even before puberty, resembling schizophrenia’s clinical course^26,55–58^. The early postnatal behavior of infant offspring in the MAM model has been only recently and incompletely reported^26,55^ with no studies explicitly examining its sequential and temporal organization.

In this study, we examined the developmental trajectory of rat vocalizations and their sensitivity to maternal potentiation, evaluating both quantitative and qualitative sound changes and their temporal and syntactic organization. As a supplementary analysis, we assessed home-seeking behavior as an early indicator of cognitive and motor functions in neonatal rats.

## Materials and methods

### Animals

Timed-pregnant Sprague–Dawley dams were obtained from Charles River, Germany, on 15th gestational day and individually housed in polycarbonate cages (22 × 38 × 18.5 cm). While this procedure may introduce transport-related stress or environmental variability, it is a widely accepted approach in MAM (methylazoxymethanol acetate) model studies^57,59–62^. To minimize potential confounds, animals were allowed a 48-h acclimatization period under standardized conditions prior to treatment.

On the 17th gestational day, dams were randomly assigned to receive an intraperitoneal (IP) injection of either vehicle (0.9% NaCl, control) or MAM (22 mg/kg, MRIGlobal, Kansas City, USA) at 1 ml/kg. No signs of distress, sickness behavior, or any visible physiological abnormalities were observed in any of the treated dams.

Pups were born 4–5 days later. No group differences were observed in the pregnancy outcome, i.e., gestational length, miscarriage rate, litter size (Fig. S1e in Supplementary Material 2), malformations, pup viability, or eye-opening. Body weight and temperature varied by cohort (Fig. S1a–d in Supplementary Material 2).

Animals were maintained under a 12-h light/dark cycle (lights on at 7 a.m.), at 21 ± 1 °C and 40–50% humidity. Behavioral testing was conducted during the light phase (8 a.m.–6 p.m.), Monday to Friday.

The experiments were conducted in accordance with the European Guidelines for animal welfare (2010/63/EU) and aligned with ARRIVE guidelines. All experimental procedures were approved by the II Local Ethics Committee for Animal Experiments at the Maj Institute of Pharmacology, Polish Academy of Science, Kraków, Poland (ethical allowance 249/2018).

### General experimental schema

**Fig. 1:**
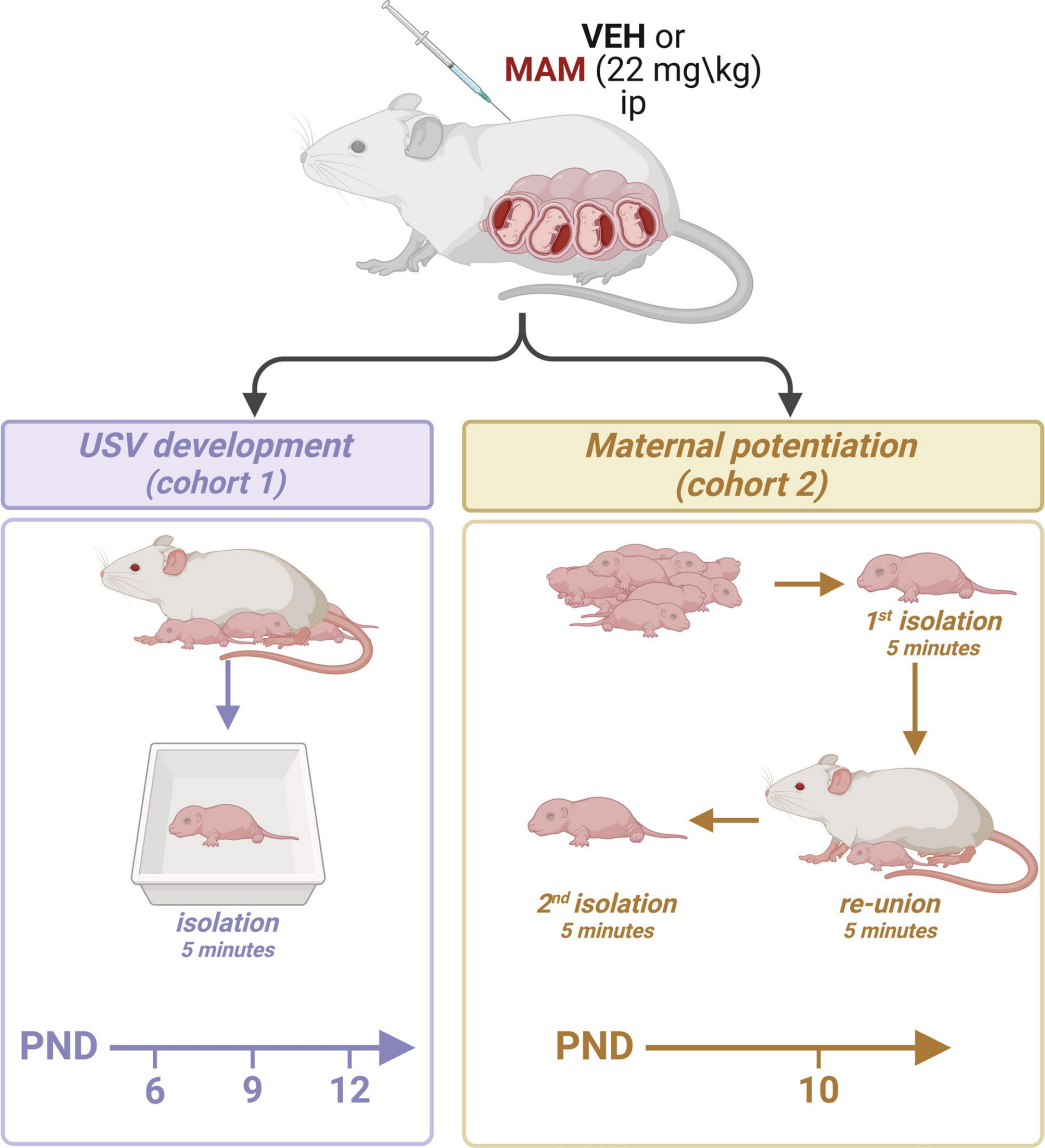
The experimental schema. VEH, vehicle (control); MAM, methylazoxymethanol acetate; PND, postnatal day. Created with BioRender.com.

The experimental schema is shown in Fig. 1. The experiments were conducted on two different cohorts of rats. The first cohort was observed to study the course of vocal development. The USVs were recorded on the 6th, 9th, and 12th PND. The second cohort was analyzed to determine the maternal potentiation effect on the 10th PND. Both male and female rats were included in all experiments. The number of animals tested in each group is summarized in Table 1.

**Table 1:** Number of rats assessed in each test.

Cohort	Procedure		VEH female	VEH male	MAM female	MAM male
1	The course of vocal development	Total number of rats	37 (− 1 excluded)	22	31	32
		Number of litters	4 (A–D)		6 (A–F)	
		Average litter size	15		11	
2	The maternal potentiation test	Total number of rats	24	35	34	24
		Number of litters	5 (E–I)		5 (G–K)	
		Average litter size	12		12	

Treatment: vehicle (VEH, control), methylazoxymethanol acetate (MAM).

### Course of vocal development (6th, 9th, 12th PND): cohort 1

Pups isolated from their mother emit ultrasonic calls indicative of a distress response^24,63^. The isolation from the mother was conducted on a total of 122 rat pups (the first cohort: four control litters A–D and six MAM litters A–F, Table 1).

The pups were recorded at three time points, i.e., on the 6th, 9th, and 12th PNDs. On the experimental day, the pups and the mother were transferred to the experimental room, where they acclimated to the new conditions for 30 min. To prevent maternal influence on pup vocalizations, the dam was removed from the home cage and placed in a separate holding cage outside the recording zone before the recording of the first pup. She remained in the holding cage throughout the entire session and was returned to the home cage only after all pups from that litter had been tested. During this time, a heating pad set to 30 ± 1 °C was placed under the litter’s cage to help maintain body temperature and prevent hypothermia. The dam’s holding area and the recording zone were separated by a sound-attenuating curtain. Pups were then taken individually from the home cage in random order and placed in a plastic container (9 × 15 × 4 cm) in a sound-attenuating styrofoam box (30 × 40 × 32 cm) for 5 min. The temperature inside the styrofoam box was equal to room temperature (21 °C ± 1 °C). After the recording, the container was thoroughly cleaned with water to minimize the effects of olfactory cues on emotional response. To ensure that testing order did not confound USV outcomes, we analyzed the relationship between pup testing sequence and USV count and found no consistent order-related effects (see Fig. S7 in Supplementary Material 2).

Body weight and temperature were assessed before the isolation period. Weight was gauged using the ED2202S scale (Sartorius AG, Germany), and temperature was measured at the back of the animal using the NC150 thermometer (Microlife AG Swiss Corporation, Widnau, Switzerland).

Pups were returned to their home cage after recording. For identification on subsequent experimental days, the pups were marked using colored, odorless markers (for details, see Table S1 in Supplementary Material 1).

### Maternal potentiation (10th PND): cohort2

A short contact with the mother temporally reduces distressed calls, and re-isolated rat pups display increased intensity of calls^33,34^.

On the 10th PND, the maternal potentiation test was conducted on 117 rat pups (the second cohort: five control litters E–I and five MAM litters G–K, Table 1). The method was adapted from Hofer et al.^64^. On the experimental day, the pups and the mother were transferred to the experimental room, where they acclimated to the new conditions for 30 min. Next, the mother was separated from the litter and placed in a holding cage (22 × 38 × 18.5 cm) outside the recording zone. Then, the pups were taken out individually from the home cage in the random order and placed in a plastic container (9 × 15 × 4 cm) that was located in a sound-attenuating styrofoam box (30 × 40 × 32 cm) for 5 min (the first isolation). To rule out order-related confounds, we analyzed the effect of testing sequence on USV output during both isolation phases and found no systematic influence of testing order (see Fig. S8 in Supplementary Material 2). The litter was kept in a home cage with a heating pad maintained at 30 ± 1 °C.

The isolated pup was transported to the dam’s holding cage upon completion of the initial isolation period (reunion). The mother and pup were left undisturbed for 5 min. The maternal behavior was recorded using a video camera (Canon LEGRIA HF R506 Handheld Camcorder 3.28 MP CMOS Full HD Black), positioned 30 cm from the side wall of the “reunion” cage and oriented toward its longer side. Behavior was manually analyzed with EthoVision XT 14 software (Nodulus Information Technology, The Netherlands). We distinguished active maternal care behaviors, including licking, grooming, and carrying^65,66^. The total duration of these behaviors and the latency to the first approach toward the pup were calculated. After the reunion period, the pups were re-isolated (the second isolation) under the same conditions as during the first isolation for an additional 5 min.

Because of the extended duration of this test (~ 15 min per animal), in contrast to the 3-min developmental USV experiment, body temperature was measured before and after the procedure to monitor potential thermoregulatory effects. Following this, the pups were returned to their home cage.

### USVs: recording and analysis

Rat pup USVs were recorded using the UltraSoundGate 116 microphone and RECORDER USGH software (version 4.2; Avisoft Bioacoustics; Glienicke/Nordbahn, Germany). The microphone was affixed to the upper lid of the styrofoam recording box ~ 25 cm from its floor.

Using the Raven Pro Interactive Sound Analysis Software (Version 1.5, The Cornell Lab of Ornithology Bioacoustics Research Program, Ithaca, NY, USA), USVs were automatically detected based on entropy analysis. The spectrograms were generated using a fast Fourier transform (FFT) length of 512 points and a 75% time-window overlap, using a 100% frame and a Hann window. Subsequently, an experimenter, blinded to the treatment, made manual adjustments (if necessary) to the detections. The Raven software produced numeric tables (CSV files) with detection windows.

The same audio and Raven’s detection CSV windows files were imported into the DeepSqueak software version 3.1.0 (on MATLAB version R2023a, The MathWorks, Inc., Natick, MA) for further analyses. This is because the DeepSqueak program offers the most comprehensive, semi-automated USV calls evaluation, including, among others, unsupervised clustering, and syntax analysis. The program created spectrograms with its default parameters using fast FFT with a window size of 0.0032 s, nfft of 0.0032 s, and a 75% overlap. Both programs provided nearly identical calls’ detection windows (data not shown).

*Basic parameters* The analyses included different call’ features, such as *the number of USVs*, *the peak frequency* (the highest frequency measured in the point of the USV characterized by the highest amplitude, in kHz), *the bandwidth* (the difference between the highest and lowest frequency, expressed in kHz regarded as a measure of frequency modulation), as well as *the duration of the call* (the length of the call measured in milliseconds).

*Temporal arrangement* According to Möhrle et al.^47^, we analyzed how MAM exposure altered the temporal structure of vocalizations (Fig. 3a). For each rat, we calculated *the inter-call interval* (the time elapsed between the end of the USV and the onset of the following USV) and classified it into three categories. The first category is the short-duration inter-call interval, which lasts at least 10 ms and separates individual USVs. The second category is the medium-duration inter-call interval, which lasted for at least 150 ms and separates the sequences of USVs. The third category is the long-duration inter-call interval, which lasted for at least 2000 ms and thus separated the bouts of USVs. These measures served mainly for the sequence analyses. In addition, we calculated *the latency to the first USV* (the time between the beginning of the recording and the start of the first USV), *the mean number of sequences, calls per sequence*, and *the mean number of bouts* and *sequences per bout*. The temporal arrangement of USV was quantified regardless of call type (cluster).

*Cluster analysis* Understanding animal vocalization has been a persistent challenge due to its complexity and high dimensionality. Comparing vocalizations across time, individuals, groups, and experimental conditions requires a method for characterizing the similarity of selected groups of vocal behaviors. We performed unsupervised clustering of USVs using the variational autoencoder (VAE) algorithm implemented in DeepSqueak version 3.1.0^67^.

Unsupervised clustering offers several advantages in audio analysis over manual labeling. It enables the automatic and objective grouping of large datasets, which is useful for the uncategorized detections. These algorithms can uncover novel, non-obvious relationships between data^68,69^. The VAE allows for compression of a single spectrogram image into a collection of 48 numbers (“latent variables”) describing data-driven vocal features. Using nearest-neighbor similarity, this "compressor” algorithm identifies latent variables that preserve a wide range of spectrogram information, effectively capturing data variability and enabling efficient categorization of similar calls (for details see^67^).

We trained the VAE model on 212,196 USVs from the vocal development experiment (6th PND: 60,305 USVs, 9th PND9th: 69,055 USVs, 12th PND: 82,836 USVs, including females and males from both VEH and MAM groups). Elbow optimization parameters were used to obtain optimal cluster numbers (Fig. S2a, b in Supplementary Material 2). The default parameters were set as follows: a maximum of 100 clusters and 3 replicates (for details of this method, see^70^). The present VEA model was then used for USV clustering in the maternal potentiation experiment.

Because visualization of a total 48-dimensional latent space is not possible, we used the Uniform Manifold Approximation and Projection for Dimension Reduction (UMAP) method^71^ to overlay and visualize clusters in a more informative and unsupervised manner (Fig. 4). UMAP is designed to create a low-dimensional graph of data that is easier to interpret while preserving the high-dimensional clusters and their relationships^69^.

We then analyzed the probabilities of transitioning between clusters for all pairs of USVs within bouts to uncover detailed calling patterns (syntax analysis). Bouts had a maximum interval of 2 s between them, consistent with the temporal arrangement analysis (see above “USVs—recording and analysis: Temporal arrangement” subsection). Transition probabilities represent the likelihood of one call type (cluster) being followed by another. The transitions with a frequency below 0.01 were excluded. Syntax analysis was conducted using DeepSqueak version 3.1.0, and the results were presented in syntax flow paths (Figs. 6, 10) as well as in the transition probability tables (Figs. S4, S6 in Supplementary Material 2). We also calculated sums across columns of transition probability tables to quantify the probability of the next call being of a certain type (Figs. 6, 10).

### Statistical analyses

The sample size was determined a priori based on an alpha (α) level of 0.05 and a statistical power of 80%. All data are expressed as means ± SEM. Outliers were identified using the Iterative Grubbs test (α = 0.05), while normality was assessed with the Kolmogorov–Smirnov test and the homogeneity of variance with Levene’s test. We tested variance sphericity using Mauchly’s test and applied Greenhouse–Geisser correction in cases of deviation. Data were analyzed using the mixed-design ANOVA (the number, basic, and temporal characteristics of USVs, and cluster distribution) or the two-way ANOVA (the maternal behavior). Physiological parameters, such as body weight and temperature, were analyzed using either mixed-design ANOVA or two-way ANOVA. Between-subject factors included treatment (VEH vs. MAM) and sex. Within-subject factors included postnatal day (6th, 9th, and 12th PND in the USV development experiment), the type of isolation (before or after reunion with mother in the maternal potentiation test), and/or the cluster (clusters 1–8 in both experiments). The percentage distribution of clusters was arcsine-transformed.

Post hoc comparisons were performed using the Newman-Keuls test. Additionally, planned comparisons were used to compare differences between first and second isolation within a given group. Data that were not normally distributed were analyzed using the non-parametric Friedman test to compare repeated measures or the Kruskal–Wallis tests to compare independent groups.

To ensure the rigor and reproducibility of our data, we controlled the litter-to-litter variation by testing the litter effect in the nested ANOVA.

The significance level was set at α = 0.05. Tables S2 and S3 (Supplementary Material 1) provides all statistical results and excluded outliers. Statistical analyses were conducted using Statistica 10.0 for Windows. Figures were generated using GraphPad Prism 10 (GraphPad Software, San Diego, CA, USA), and cluster as well as syntax analyses and their visualizations were performed in DeepSqueak 3.1.0 running on MATLAB (version R2023a, The MathWorks, Inc., Natick, MA).

## Results

The results summary is provided in Table S5 (Supplementary Material 1).

### Course of USVs development (6th, 9th, and 12th PND): cohort 1

As the USV recording for one of the control females at the 9th PND was missing due to technical problems, the missing values were replaced with the mean of the intact values in the same group^72^. Additionally, one of the control females was excluded from the analysis due to the lack of USV on the 12th PND (for a total number of animals, see Table 1).

#### MAM treatment reduces the number of USVs across postnatal development

**Fig. 2:**
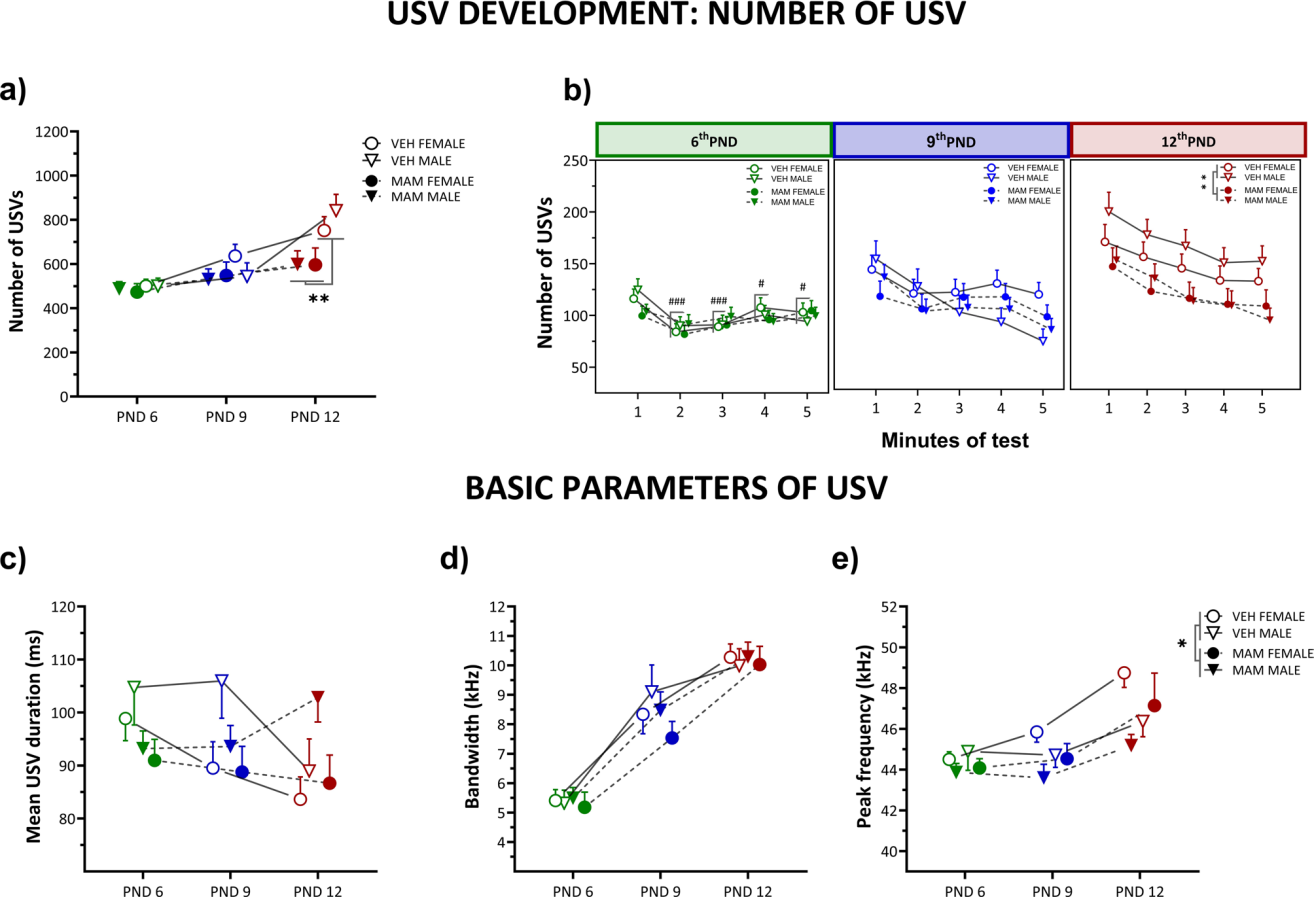
MAM treatment reduces the number and alters the basic parameters of isolation-induced USVs across postnatal development. (**a**) Total number of USVs recorded on 6th, 9th, and 12th postnatal days for control and MAM-treated female and male pups. On the 12th postnatal day, MAM-treated pups produced fewer USVs than controls. (**b**) Temporal pattern of USVs across the 5 min of testing. On the 6th postnatal day, control, but not MAM-treated pups produced more USVs during the first minute of recording than in the subsequent minutes. On the 12th postnatal day, MAM-treated pups had fewer USVs than controls throughout the recording. (**c**) Control pups displayed shorter USV durations at later postnatal days, while MAM-treated pups maintained relatively stable durations over time. Males of both treated groups produced longer calls than females. (**d**) The USV bandwidth increased over time in control and MAM-treated pups. (**e**) Although peak frequency increased with age in both control and MAM-treated pups, MAM-treated pups exhibited consistently lower peak frequencies than controls, regardless of age. Regardless of the treatment, male rats vocalized at a lower frequency than females. Points represent the mean ± S.E.M. Statistical significance is indicated as follows: *p < 0.05; **p < 0.01 (**a**, **e**) vs. control, #*p* < 0.05; ###*p* < 0.001 vs. first minute for controls (**b**).

As the control pups were aging, we observed an increase in the emission of USVs (12th PND vs. 6th PND and 9th PND: *p* < 0.001 for both comparisons; Fig. 2a, Table S2). In contrast, MAM pups produced a stable number of USVs across all testing days. On the 12th PND, when USV emission peaked in controls, MAM-exposed pups of both sexes emitted fewer USVs than controls (MAM vs. control pups at the 12th PND: *p* < 0.01; Fig. 2a, Table S2). The increasing number of isolation-induced calls in control animals may reflect growing sensitivity to separation and maturation of social communication.

Sex does not affect the number of emitted USVs.

The minute-by-minute analysis suggests that control pups show an initial peak in USV production on the 6th PND, which is absent in MAM-treated pups, indicating early impairments in vocalization dynamics (Fig. 2b, Table S2). By the 12th PND, MAM pups consistently emit fewer USVs than controls, suggesting persistent deficits in social communication or distress signaling (*p* < 0.01, Fig. 2b).

The number of emitted USVs did not differ between litters in a particular group (Fig. S3 in Supplementary Material 2, Table S2).

#### The basic characteristics of USV are affected by MAM

*Mean USVs duration* The duration of control pups’ USVs decreased over consecutive PNDs. They emitted shorter calls on the 12th PND than the 6th PND (12th PND vs. 6th PND for control pups: *p* < 0.01; Fig. 2c, Table S2). In contrast, MAM-treated pups exhibited stable USV durations across PNDs (Fig. 2c, Table S2). This pattern may indicate an effective maturation of vocalization control in typically developing but not MAM-treated pups.

In addition, regardless of treatment and PND, males produced longer calls than females did (*p* < 0.05; Fig. 2c, Table S2).

*Bandwidth* The USV bandwidth, a proxy measure of the complexity of ultrasonic communication, increased as the pups matured (Fig. 2d, Table S2). On the 9th and 12th PNDs, all pups emitted USVs with a broader bandwidth than the 6th PND (*p* < 0.001 for both comparisons), with the 12th PND USVs being wider than those on the 9th PND (*p* < 0.001). While MAM rats appeared to produce USVs with a narrower bandwidth, particularly on the 9th PND, these changes were not statistically significant. This parameter was also not affected by the sex.

*Peak frequency* Over time, the USV’s peak frequency increased in all rats (12th PND vs. 6th PND and 9th PND: *p* < 0.001; Fig. 2e, Table S2). Although both treatments resulted in similar patterns of frequency changes, the MAM pups, regardless of age, emitted calls at a lower frequency (*p* < 0.05; Fig. 2e, Table S2). As high peak frequency may indicate effective stress management, the lower frequency in MAM pups suggests impaired distress regulation and potential neurodevelopmental deficits.

Regardless of the treatment, male rats vocalized at a lower frequency than females (*p* < 0.05; Fig. 2e, Table S2).

#### The temporal pattern of USVs differed between the MAM and control groups

**Fig. 3:**
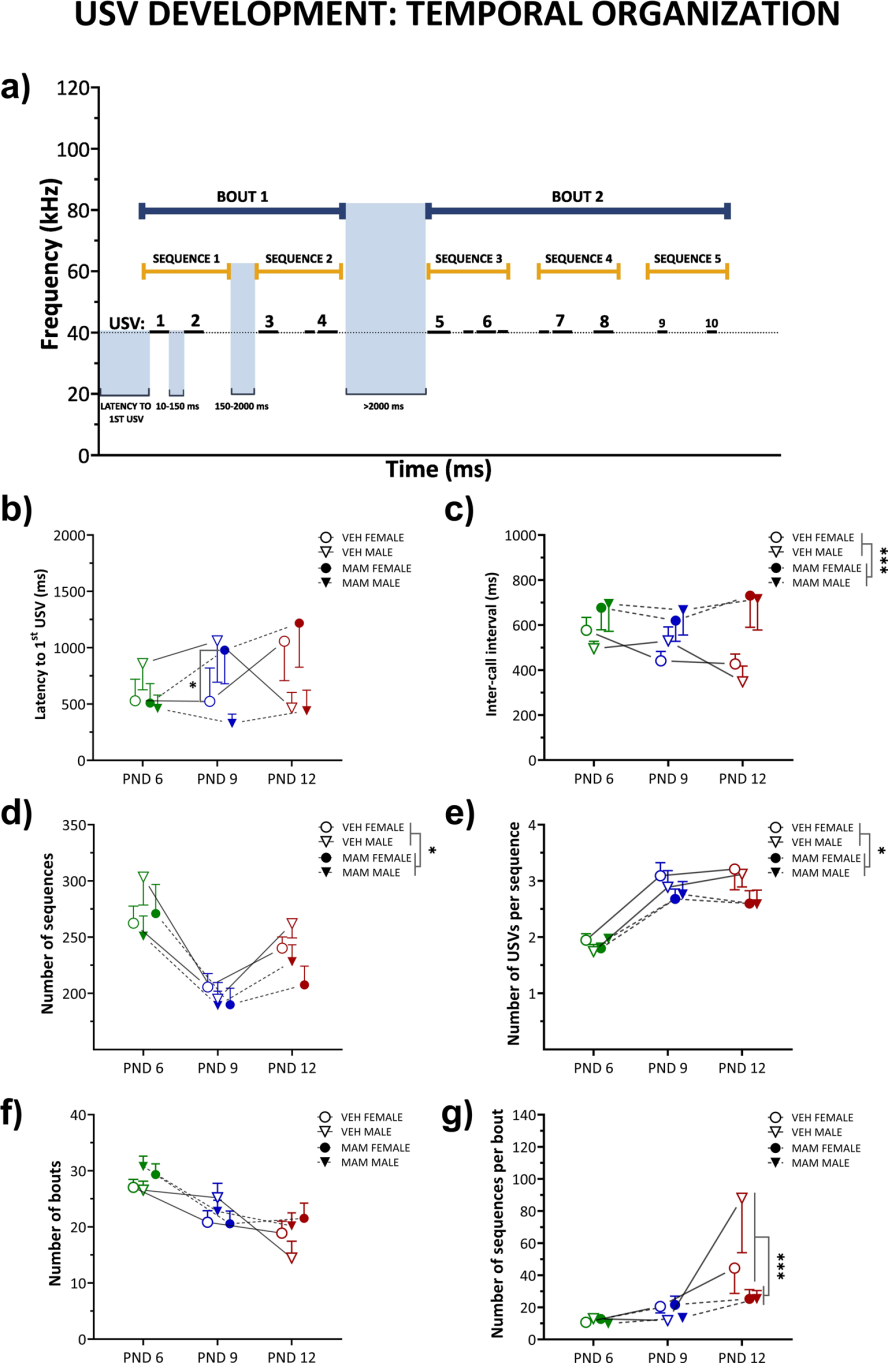
MAM treatment affects the temporal organization of isolation-induced USVs across postnatal development. (**a**) Schematic representation of the temporal organization of USVs (black horizontal lines). Latency to the first USV (the time from the start of the recording to the beginning of the first USV), inter-call interval (the mean gap between USVs), sequences (defined as groups of USVs separated by medium,150–2000 ms, inter-call interval), and bouts (groups of sequences separated by long, ≥ 2000 ms, inter-call interval) are illustrated. (**b**) Latency to the first USV remained comparable across postnatal days. MAM female pups showed longer latency than control females by the 9th postnatal day. (**c**) The mean duration of the inter-call interval remained comparable across postnatal days; however at 6th–12th postnatal days, MAM-treated pups displayed longer inter-call intervals than controls. (**d**) The number of USV sequences was the highest on the 6th postnatal day and the lowest on the 9th postnatal day. Irrespective of postnatal day, the number of USV sequences was lower in MAM-treated pups than in controls. (**e**) The mean number of USVs per sequence increased with the age of the animals. Irrespective of postnatal day, MAM-treated pups produced fewer USVs per sequence than control pups. (**f**) The number of USV bouts decreased with each postnatal day. MAM-treated pups displayed a similar number of bouts to control pups across all postnatal days. (**g**) The number of USV sequences per single bout increased with the age of control but not MAM-treated pups. MAM-treated pups produced fewer sequences per bout than controls at the 12th postnatal day. Points represent the mean ± S.E.M. Statistical significance is indicated as follows: *p < 0.05; **p < 0.01; ***p < 0.001 vs. control.

Figure 3a illustrates the temporal organization scheme of the infant’ USVs adapted from Möhrle et al.^47^.

*Latency to 1st USVs* The latency to vocalization onset was comparable across successive PNDs for all experimental groups. The only difference appeared on the 9th PND, with MAM-treated female pups showing a longer delay before their first USV compared to controls (*p* < 0.05; Fig. 3b, Table S2).

*Inter-call interval * The duration of the inter-call interval stayed consistent across PNDs in all groups. Every PND, MAM-treated pups made longer pauses between USVs than controls (*p* < 0.001; Fig. 3c, Table S2). There were no differences between males and females on the inter-call interval duration.

*The number of sequences* In all animals, the number of USV sequences was the highest on the 6th PND and the lowest on the 9th PND (6th PND vs. 9th PND and 12th PND: *p* < 0.001, 9th PND vs. 12th PND: *p* < 0.001; Fig. 3d, Table S2). Irrespective of the PND, the number of USV sequences was lower in MAM animals (*p* < 0.05; Fig. 3d, Table S2). This parameter was not influenced by sex.

*The number of USVs per sequence* The mean number of USVs per sequence increased with age in controls (6th PND vs. 9th PND and 12th PND: *p* < 0.001 for both comparisons), but remained lower and age-independent in MAM-treated pups (Fig. 3e, Table S2). This parameter was not influenced by sex.

*The number of bouts* The number of USV bouts decreased with each PND in all animals (6th PND vs. 9th PND and 12th PND: *p* < 0.001 for both comparisons, 9th PND vs. 12th PND: *p* < 0.05; Fig. 3f, Table S2). The MAM pups did not differ from the control group in this measure. This parameter was also not influenced by sex.

*The number of sequences per bout* The number of USV sequences per single bout increased with the age of control but not MAM-treated pups (12th PND vs. 6th PND and 9th PND for control pups: *p* < 0.001 for both comparisons). On the 12th PND, the MAM animals displayed fewer sequences per bout than the controls (*p* < 0.001; Fig. 3g, Table S2). This parameter was not influenced by sex.

Overall, data presented in Fig. 3 suggest lower efficacy of separation calls and diminished call’ complexity due to the MAM treatment.

#### The call type profile is altered in MAM pups

**Fig. 4:**
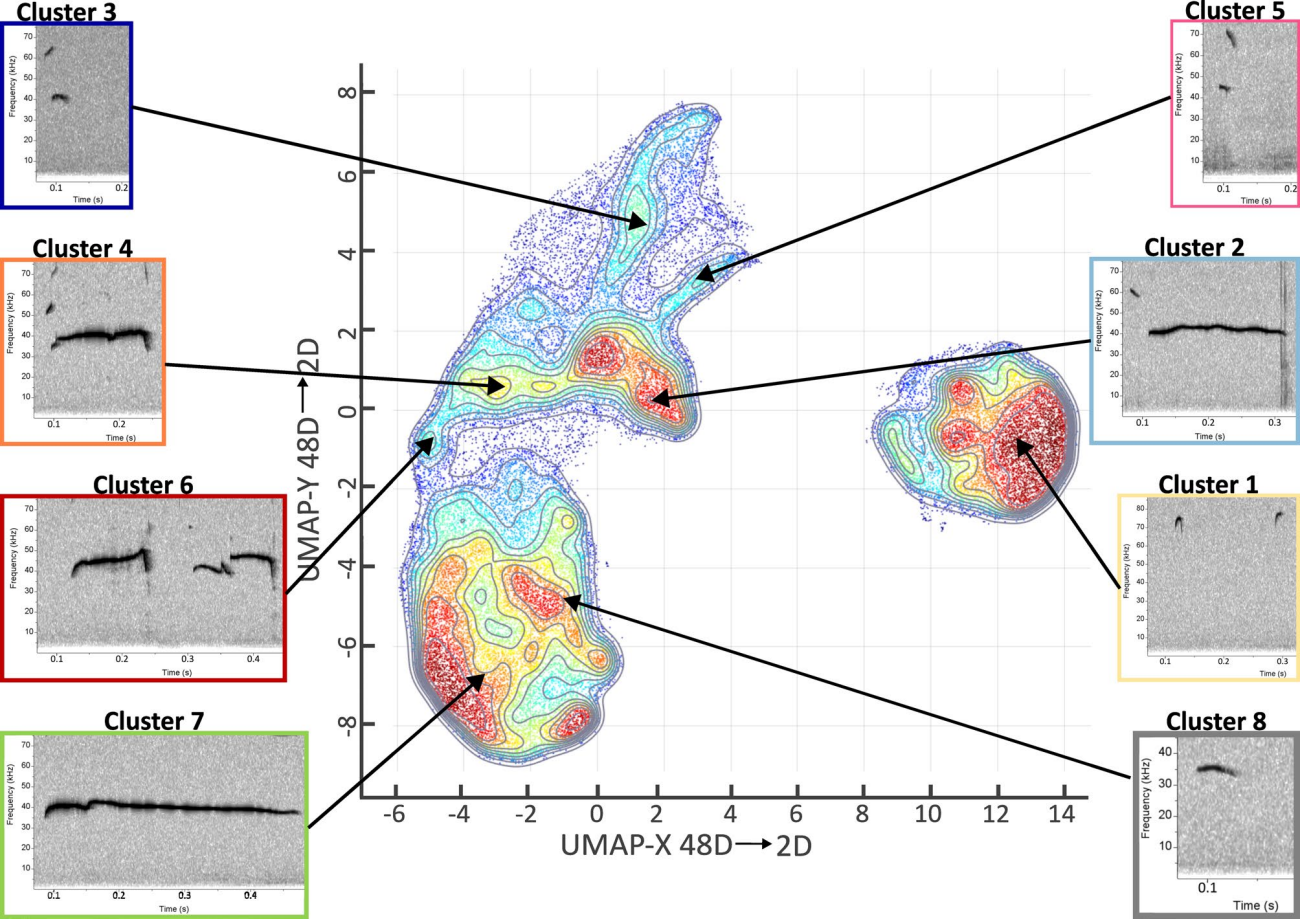
Clustering of ultrasonic calls in pup-isolated rats using variational autoencoder and density-based UMAP visualization. The UMAP representation illustrates high-dimensional USVs projected onto two dimensions with density overlays. The plot displays distinct USV clusters labeled 1 to 8, each associated with miniature spectrograms representative of the cluster’s vocalization features. Each cluster corresponds to a specific USV subtype characterized by unique temporal and spectral properties. The axes represent the reduced-dimensionality coordinates, while contour lines indicate data density, with color gradients transitioning from high density (red) to low density (blue). This topological plot visualizes USV clusters for isolated control pups recorded on the 12th PND, including 22 males and 37 females, with a total of 45,582 vocalizations.

*Clusters’ distribution:* The VEA unsupervised clustering algorithm grouped USVs into 23 clusters (Fig. S2a, b in Supplementary Material 2), purportedly representing unique calls’ types. Some clusters showed minimal differences in basic USV features, e.g., mean duration, bandwidth, and peak frequency (Table S4 in Supplementary Material 1). Thus, we merged similar clusters by analyzing these features and using the UMAP projection for the 12th PND, where the diversity of USVs was highest (Fig. 4).

We identified eight topologically distinct clusters, that could be characterized as (1) high-pitched short USVs; (2), (3), and (4): step-down USVs with instantaneous frequency change to a lower frequency; (5) step-up USVs with instantaneous frequency change to a higher frequency; (6) frequency-modulated USVs; (7) flat USVs; and (8) low-frequency short USVs (see Table 2 for detailed characteristics).

**Table 2:** Clusters characteristic.

Cluster name	General characteristic
Cluster 1	Very high peak frequency: > 50 kHz Very short call duration: < 20 ms Narrow bandwidth: < 8 kHz Slope: 35–82 and > 440 (kHz/s)
Cluster 2	Very low peak frequency: < 41 kHz Long call duration: > 100 ms Wide bandwidth: 15–25 kHz Medium slope: − 26 to (− 115) kHz/s
Cluster 3	Medium peak frequency: < 50 kHz Low call duration: ~ 40 ms Very wide bandwidth: > 25 kHz Very high slope: < − 600 kHz/s
Cluster 4	Medium peak frequency: < 50 kHz Long call duration: 50–150 ms Wide bandwidth: > 15 kHz Medium slope: − 190 to (− 17) kHz/s
Cluster 5	Medium peak frequency: ~ 50 kHz Medium call duration: 40 ms Very wide bandwidth: > 25 kHz High Slope: > 400 kHz/s
Cluster 6	Medium peak frequency: < 50 kHz Long call duration: ~ 150 ms Wide bandwidth: > 8 kHz Low slope: 18 kHz/s
Cluster 7	Very low peak frequency: < 40 kHz Long call duration: 75–155 ms Narrow bandwidth: < 7kHz Medium slope: − 35 to 125 kHz/s
Cluster 8	Low peak frequency: < 45 kHz Low call duration: < 40 ms Very narrow bandwidth: < 4 kHz Low slope: − 44 to 17 kHz/s

Parameters calculated based on AVG value for all PNDs (6th–12th) for control and MAM-treated females and males.

**Fig. 5:**
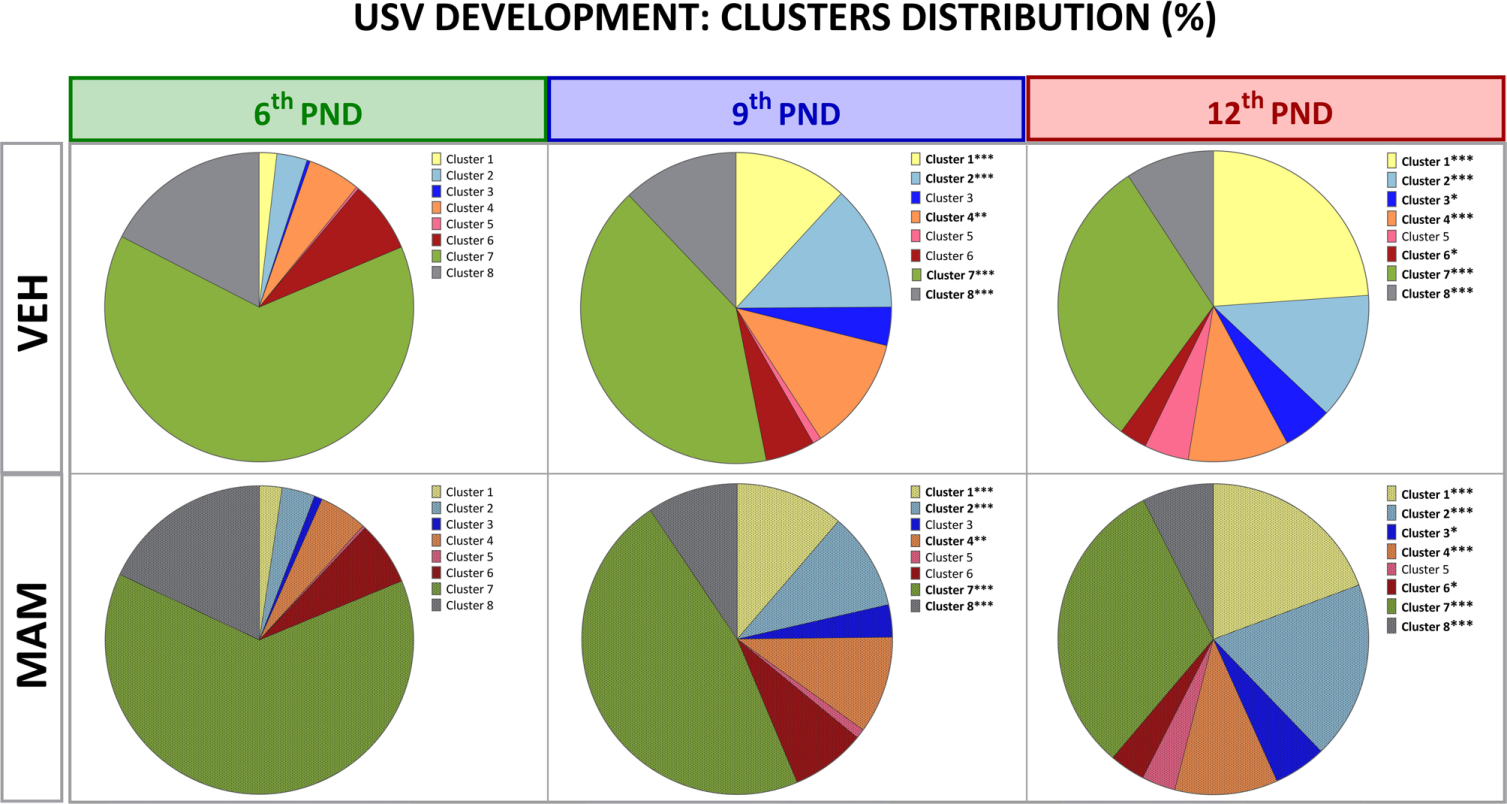
Effect of prenatal MAM exposure on the distribution of isolation-induced USV clusters across postnatal development. Pie charts represent the percentage distribution of USV clusters in control (clear figures) and MAM-treated pups (dotted figures) across the 6th, 9th, and 12th postnatal days. Female’s and male’s results were pooled. Each cluster corresponds to a distinct vocalization pattern categorized by specific acoustic parameters. Prenatal MAM exposure did not affect cluster distribution. In control and MAM-treated animals, the low-pitch flat cluster 7 and low-pitch short cluster 8 dominated the distribution on the 6th postnatal day. By the 9th and 12th postnatal days, the proportion of clusters 7 and 8 decreased while the contributions of other clusters increased. These included the high-pitched short cluster 1, low-pitched, long “step-down” cluster 2, medium-pitched, short, very wide “step-down” cluster 3, medium-pitched, medium-duration, medium-bandwidth “step-down” cluster 4, and “frequency-modulated” cluster 6. Statistical significance is indicated for differences between the 6th and the 9th/12th postnatal day: *p < 0.05; **p < 0.01; ***p < 0.001.

The low-pitched, flat cluster 7 was the most common vocalization in rat pups (Fig. 5, Table S2). As animals aged, their vocalizations become more complex. Regardless of the treatment, the incidence of cluster 7 and low-pitched, short cluster 8 calls decreased gradually with the age of animals (cluster 7—6th vs. 9th and 12th PND: *p* < 0.001 for both comparisons, cluster 8—6th vs. 9th and 12th PND: *p* < 0.001 for both comparisons; Fig. 5, Table S2). In turn, the usage of high-pitched short cluster 1 or step-down clusters 2 and 4 calls increased on the 9th and 12th PND (cluster 1—6th vs. 9th and 12th PND: *p* < 0.001 for both comparisons, cluster 2—6th vs. 9th and 12th PND: *p* < 0.001 for both comparisons, cluster 4—6th vs. 9th PND: *p* < 0.01, 6th vs. 12th PND: *p* < 0.001; Fig. 5). Additionally, on the 12th PND, cluster repertoire has increased by step-down clusters 3 (6th vs. 12th: *p* < 0.01) and frequency-modulated cluster 6 (6th vs. 12th: *p* < 0.05; Fig. 5). Neither treatment nor sex affected the call type profile.

Overall, these analyses suggest that along with the development, the animals, regardless of the treatment, displayed more complex, high-pitched calls, i.e., represented by all clusters except cluster 7 (flat calls) and cluster 8 (short calls of low frequency).

*Syntax analysis* It has been shown that different types of USVs are not randomly ordered. Instead, they are organized in phrases and motifs^30,47^. We attempted to elucidate the complexity of all cluster transitions within USV bouts during pups’ development and how MAM exposure affected these syntax flows. For this analysis, USVs from males and females were pooled since there were no main effects or interactions of sex on cluster distribution within any of the groups (Table S2 in Supplementary Material 1).

**Fig. 6:**
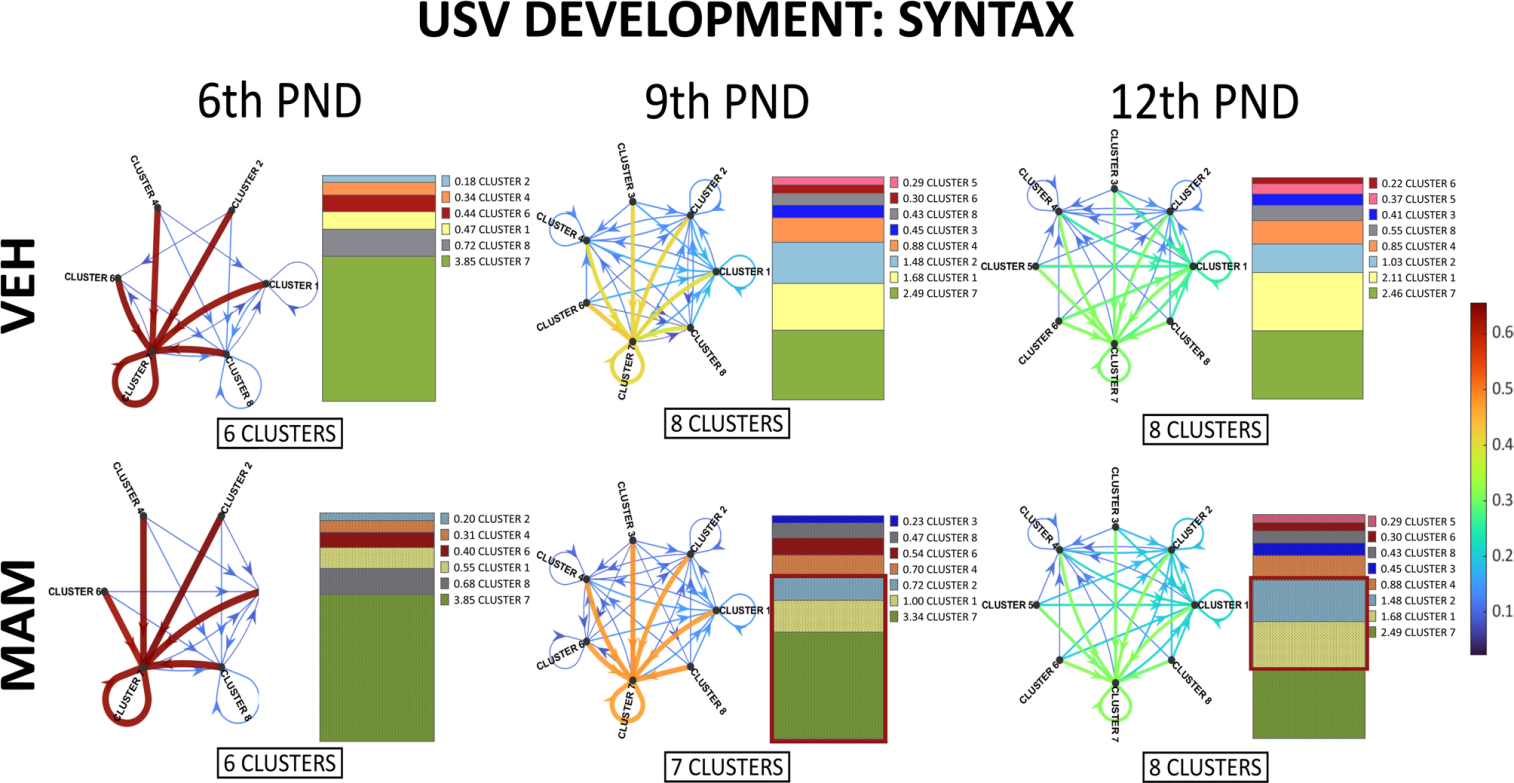
MAM treatment affects conditional probability for each cluster transition within USV bouts across postnatal development. The transitioning between call types in and MAM pups on the 6th, 9th, and 12th postnatal days is displayed in a syntax flow path representing the conditional probability of USVs changing from one call type to another. Arrows represent the directions of transitions. Thicker arrows and warmer colors denote higher transition probabilities. A probability below 0.075 was not shown for syntax flow path graph clarity. Vertical sliced bars represent summed conditional probabilities for cluster transitions within USV bouts in and MAM pups on the 6th, 9th, and 12th postnatal days. Summed transition probabilities for the next cluster are sorted from the lowest to highest within the treatment and postnatal days. On the 9th postnatal day, MAM-treated pups were more likely to transition to cluster 7 but less likely to clusters 1 and 2 than controls. On the 12th postnatal day, they showed a higher probability of transitioning to cluster 2 and a lower probability to cluster 1.

On the 6th PND, control and MAM-treated pups transitioned between six clusters (Figs. 6, S4 in Supplementary Material 2). Two clusters (3 and 5) had transition probabilities below 0.01 and were therefore excluded from the analysis (see the “USVs—recording and analysis” section). Cluster 7 was the most frequently transitioned, followed by clusters 8 and 1, including repetitive use. As the pups aged, their USV syntax diversity increased. By the 9th PND and the 12th PND, the number of clusters increased to seven or eight, and the order of the most frequently transitioned clusters shifted (Figs. 6, S4). While cluster 7 remained the most frequently transitioned, its transition frequency decreased daily. On the 9th PND and the 12th PND, clusters 1 and 2 replaced cluster 8 as the next most frequently transitioned clusters.

Compared to the controls, on the 9th PND, MAM-treated pups were more likely to transition to cluster 7 but less likely to clusters 1 and 2 (Figs. 6, S4 in Supplementary Material 2). The slight differences were also found on the 12th PND as MAM pups displayed a higher probability of transitioning to cluster 2 and a lower probability of transitioning to cluster 1 than the control (Figs. 6, S4 in Supplementary Material 2).

Overall, these analyses suggest the development of increasingly complex “syntax” of the calls in control animals and somewhat less complicated syntax in the impaired pups.

### Maternal potentiation test (10th PND): cohort 2

#### MAM exposure weakened the maternal potentiation effect

**Fig. 7:**
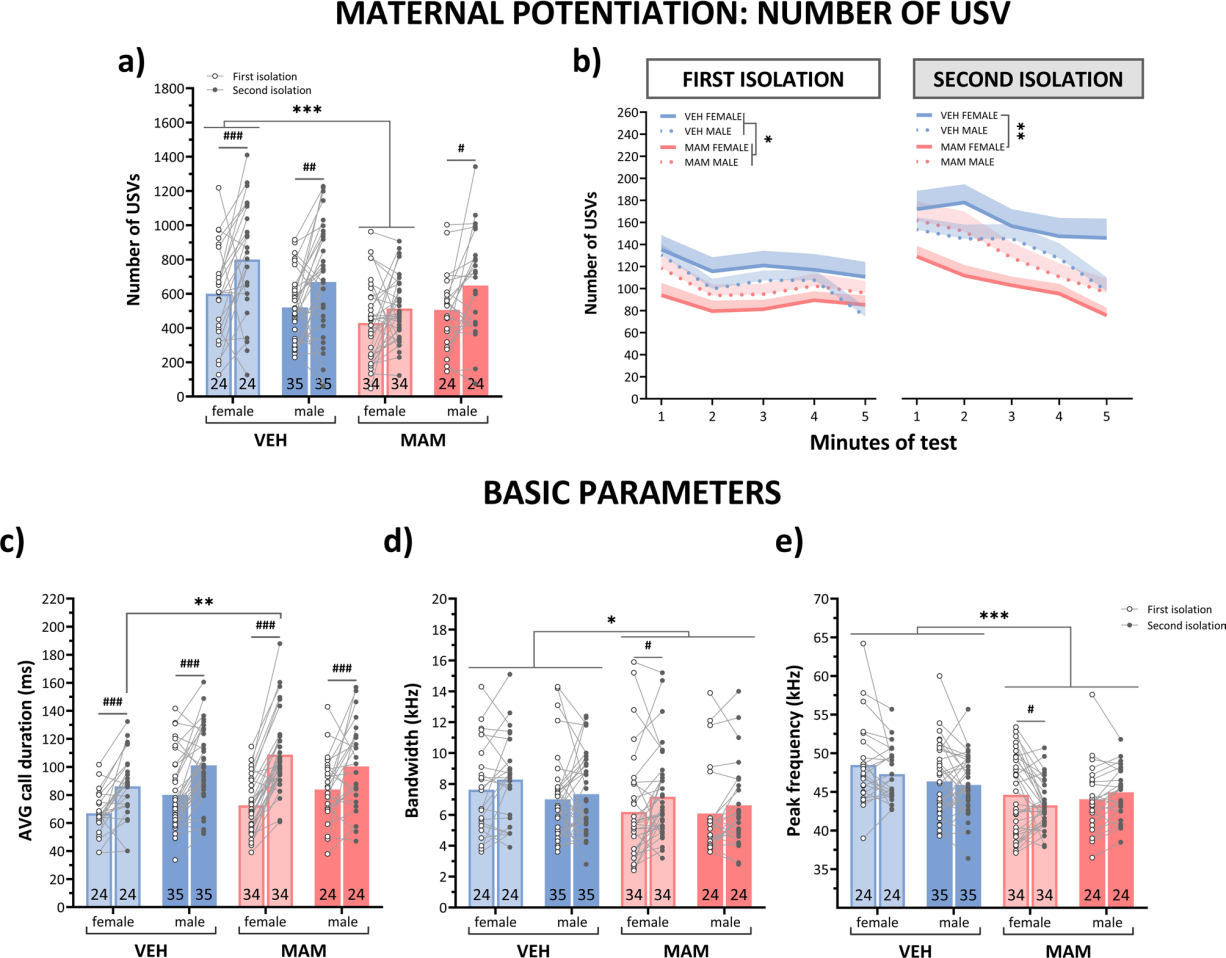
MAM treatment reduces the number and alters the basic parameters of isolation-induced USVs in the maternal potentiation test. (**a**) Total number of USVs recorded during the first isolation (before maternal contact) and the second isolation (after maternal contact) for control and MAM-treated female and male pups. Maternal contact increased USV production in the re-isolated control pups. This effect was absent in MAM-treated female pups and less pronounced in MAM-treated male pups. MAM-treated female pups produced fewer USVs than control females in both isolation periods. (**b**) Number of USVs recorded per minute during the first and second isolation tests. MAM-treated female pups produced fewer USVs than controls in each minute of both isolation periods. MAM-treated male pups produced fewer USVs than control males only during the first isolation test. In both groups, USV production gradually declined across the 5-min duration of each test. (**c**) Maternal contact increased the mean call duration in re-isolated control and MAM-treated pups. During the second isolation, MAM-treated females produced longer USVs than control females. (**d**) Maternal contact increased the bandwidth in re-isolated MAM-treated females. Regardless of the isolation period, MAM-treated pups vocalized with flatter USVs. (**e**) Maternal contact decreased the peak frequency in re-isolated MAM-treated female pups. Additionally, MAM-treated pups exhibited lower peak frequencies than controls across both isolation conditions. Points represent the mean ± S.E.M. Statistical significance is indicated as follows: *p < 0.05; **p < 0.01; ***p < 0.001 versus control, #*p* < 0.05; ##*p* < 0.01; ###*p* < 0.001 versus first isolation. The annotations at the bottom of the bar indicate the sample size.

The control pups demonstrated a maternal potentiation effect, because following the contact with the dam, the re-isolated pups emitted a greater number of USVs compared to their initial isolation (first vs. second isolation for control females: *p* < 0.001, for control males: *p* < 0.01; Fig. 7a, Table S3). This effect was absent in MAM-treated female pups, and it was weak in MAM males (first vs. second isolation: *p* < 0.05; Fig. 7a, Table S3). Since increased re-isolation calls could be regarded as an indication of a mother–pup relationship, MAM-induced attenuation or absence of this effect may suggest a deficit in the development of the maternal bond.

In addition, regardless of the type of isolation, MAM female, but not male, pups produced a lower number of USVs than controls (MAM-treated vs. control females: *p* < 0.001; Fig. 7a, Table S3).

Across recordings, pups produced fewer calls over time, regardless of treatment. During initial isolation, MAM-treated pups consistently emitted fewer USVs than controls each minute (MAM vs. control pups: *p* < 0.05; Fig. 7b, Table S3). During re-isolation, only MAM-treated females showed reduced USV emission in every minute of the session (MAM vs. control females: *p* < 0.01; Fig. 7b, Table S3). These observations are consistent with the first experiment’s results, demonstrating the decreased number of isolation-induced calls over time of recording at 12th PND in a different cohort of rats (Fig. 2).

The number of emitted USVs did not differ between litters in a given treatment group (Fig. S5 in Supplementary Material 2, Table S3).

##### The basic characteristics of USV are affected by MAM

*Mean USVs duration* After maternal contact, both control and MAM-treated pups increased USV duration upon re-isolation compared to initial isolation (the first vs. second isolation for all groups: *p* < 0.001; Fig. 7c, Table S3). While USV duration was similar between groups during initial isolation, re-isolated MAM-treated females, but not males, emitted longer USVs than controls (*p* < 0.01; Fig. 7c, Table S3).

During initial isolation, males in both treatment groups produced longer USVs than females (males vs. females during the first isolation: *p* < 0.05), but this sex difference was not detectable during reisolation (Fig. 7c, Table S3).

*Bandwidth* Comparing initial and second isolation, only MAM-treated female pups showed increased call bandwidth upon re-isolation (first vs. second isolation for MAM females: *p* < 0.05; Fig. 7d, Table S3). Regardless of the isolation, MAM-treated pups vocalized with the flatter USVs (*p* < 0.05; Fig. 7d, Table S3).

Sex did not affect the call bandwidth in any isolation period (Fig. 7d, Table S3).

*Peak frequency* Planned comparisons revealed that only MAM-treated female pups, when re-isolated, emitted USVs with reduced peak frequency (first vs. second isolation for MAM females: *p* < 0.05; Fig. 7c, Table S3).

Regardless of isolation periods, MAM-treated pups produced USVs of lower frequency than controls (*p* < 0.001; Fig. 7c, Table S3), and this effect was common for both sexes. Overall, these analyses suggest that MAM treatment alters the acoustic characteristics of USVs, particularly in females.

#### The temporal pattern of USVs differs between the MAM and control groups

**Fig. 8:**
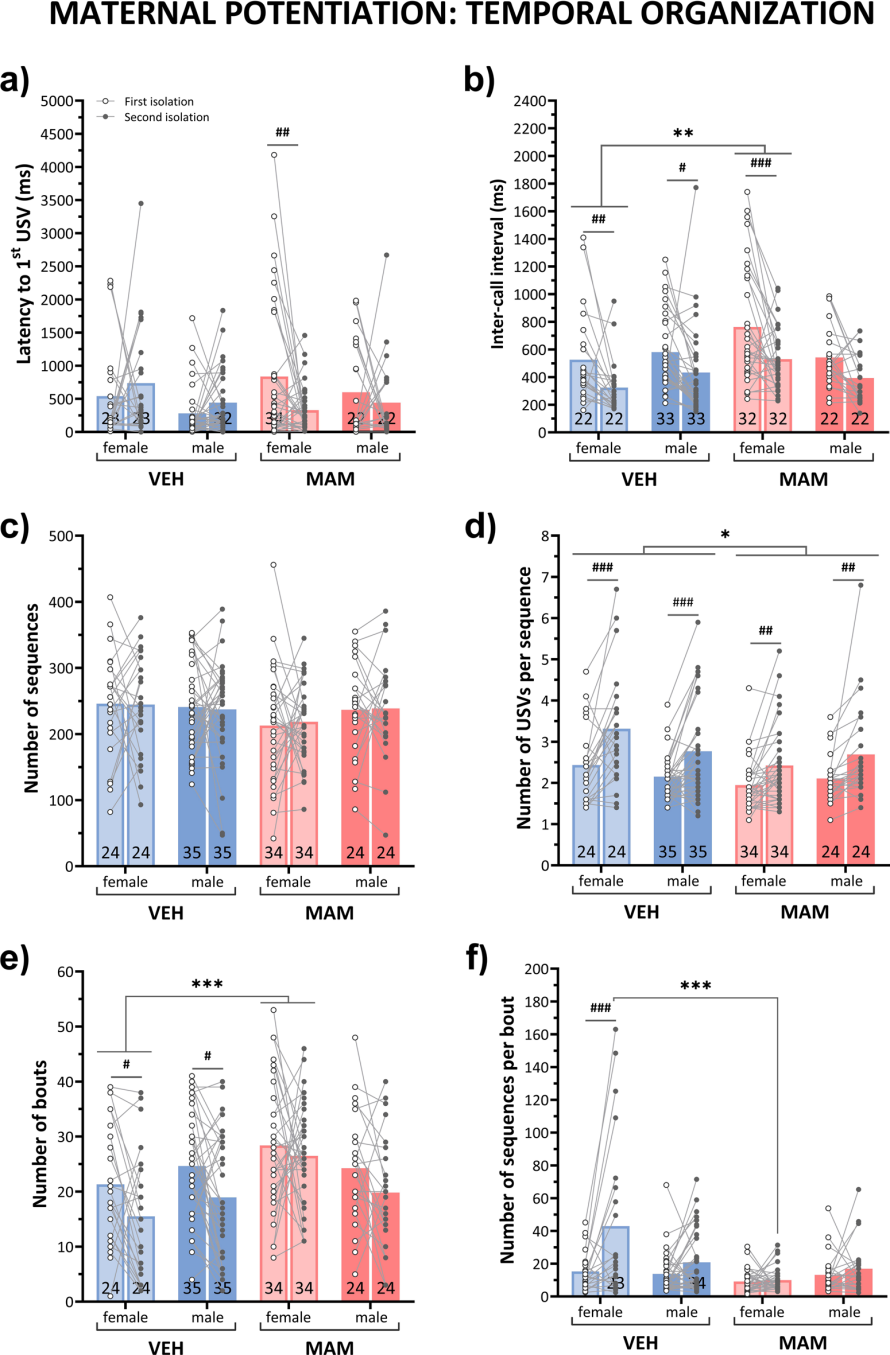
MAM treatment affects the temporal organization of isolation-induced USVs in the maternal potentiation test. (**a**) During the first isolation test, MAM-treated animals began vocalizing later than controls. During the second isolation, unlike controls, MAM-treated animals started vocalizing earlier compared to their initial isolation. (**b**) During re-isolation, the inter-call interval decreased in control females, control males, and MAM-treated females but not in MAM-treated male pups. Regardless of the isolation period, MAM-treated female pups exhibited longer inter-call intervals compared to controls. (**c**) Neither maternal contact nor treatment or sex affects the number of USV sequences. (**d**) During re-isolation, the number of USVs per sequence increased in control and MAM-treated pups. Irrespective of the isolation period, MAM-treated pups displayed fewer USVs per sequence than control pups. (**e**) During re-isolation, the number of bouts was lower in control pups but not in the MAM-treated pups. Irrespective of the isolation periods, MAM-treated female pups demonstrated more USV bouts than control females. (**f**) During re-isolation, the number of sequences per bout increased in control females but not in other groups. Regardless of the isolation period, MAM-treated females exhibited fewer sequences per bout than control females. Points represent the mean ± S.E.M. Statistical significance is indicated as follows: *p < 0.05; **p < 0.01; ***p < 0.001 vs. control, ***p < 0.001 versus control, #*p* < 0.05; ##*p* < 0.01; ###*p* < 0.001 vs. first isolation. The annotations at the bottom of the bar indicate the sample size.

*Latency to first USVs* The time to vocalization onset differed between the first and second isolations only in MAM-treated females, who began vocalizing earlier during re-isolation (first vs. second isolation for MAM females: *p* < 0.01; Fig. 8a, Table S3). In turn, in the initial isolation, MAM-treated pups vocalized later than controls (MAM vs. control pups for the initial isolation: *p* < 0.05; Fig. 8a, Table S3).

There were no overall sex differences in the latency to initiate vocalization (Fig. 8a, Table S3).

*Inter-call interval duration* Maternal contact significantly shortened the inter-call interval in re-isolated animals (first vs. second isolation: *p* < 0.001). Particularly, the inter-call interval decreased in re-isolated control females (*p* < 0.01), control males (*p* < 0.05), and MAM females (*p* < 0.001), but not in MAM males (Fig. 8b).

Additionally, independent of the isolation period, MAM-treated female pups exhibited longer inter-call intervals than control females (MAM vs. control females: *p* < 0.01; Fig. 8b, Table S3).

*The number of sequences* The number of call sequences was comparable between isolation periods, treatment groups, and sexes (Fig. 8c, Table S3).

*The number of USVs per sequence* The number of USVs per sequence increased after contact with the mother in all re-isolated control and MAM-treated pups; however, this effect was weaker in MAM-treated rats (control females: *p* < 0.001, control males: *p* < 0.001, MAM females: *p* < 0.01, MAM males: *p* < 0.01; Fig. 8d, Table S3). Regardless of the isolation period, this parameter was lower in MAM-treated females and males (*p* < 0.05; Fig. 8d, Table S3).

There were no differences between the sexes for this parameter (Fig. 8d, Table S3).

*The number of bouts* The number of USV bouts decreased after maternal contact in control pups, but remained unchanged in MAM-treated pups (control females: *p* < 0.05, control males: *p* < 0.05; Figure . 8e, Table S3).

Regardless of the isolation period, MAM-treated females, but not males, exhibited more USV bouts than controls (MAM females vs. control females *p* < 0.001; Fig. 8e, Table S3).

*The number of sequences per bout* The number of USV sequences per bout increased after maternal contact only in control females (first vs. second isolation for control females: *p* < 0.001; Fig. 8f, Table S3). Following re-isolation, MAM females exhibited fewer USV sequences per bout compared to controls (*p* < 0.001), while no differences were observed between MAM and control males. During the initial isolation, there were no significant differences between treatments or sexes in the number of USV sequences per bout (Fig. 8f, Table S3).

Overall, these analyses suggest that MAM treatment disrupts the normal vocalization patterns, particularly in females, leading to altered latency, inter-call intervals, bout structure, and call sequencing. These results are consistent with the vocalization development experiment shown at Fig. 3.

#### The call type profile is altered in MAM pups

**Fig. 9:**
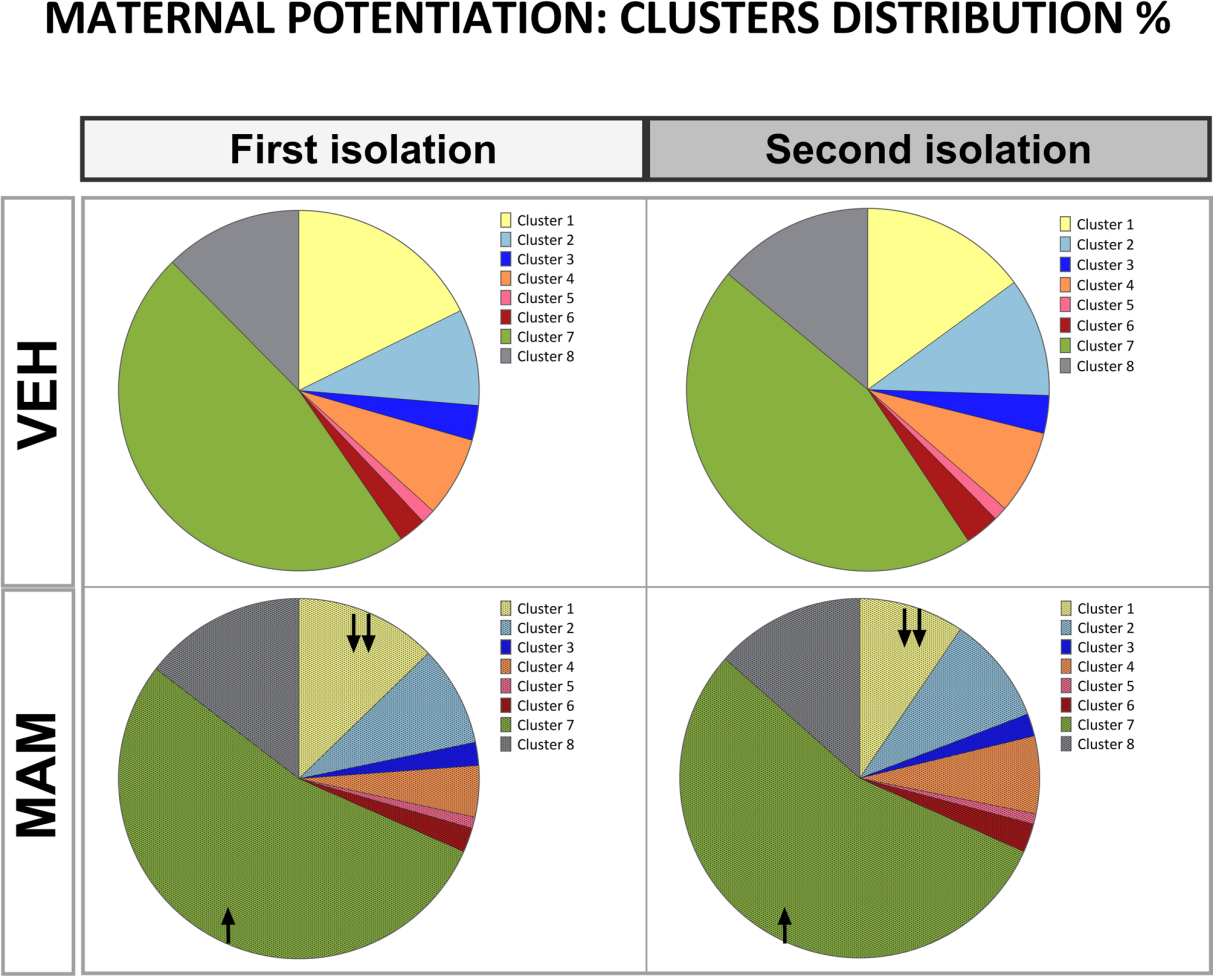
MAM treatment affects the distribution of USV clusters in the maternal potentiation test. Pie charts represent the percentage distribution of USV clusters in control (clear figures) and MAM-treated pups (hatched figures) during the first isolation (before maternal contact) and the second isolation (after maternal contact). Female and male results were pooled. Each cluster corresponds to a distinct vocalization pattern categorized by specific acoustic parameters. Across both isolation periods, MAM-treated pups, compared to control, showed a lower proportion of low-pitched flat cluster 7 and a higher proportion of high-pitched short cluster 1 (Statistical significance is indicated for differences between the control and MAM-treated pups: ↑*p* < 0.05; ↓↓*p* < 0.01).

*Call type distribution* Contact with the mother affects the distribution of the clusters. During the second isolation, the low-pitched flat cluster 7 increased, while the high-pitched short cluster 1 decreased compared to the first isolation (cluster 7: *p* < 0.01, cluster 1: *p* < 0.001; Fig. 9, Table S3), showing a general pattern of change for all animals.

Across both isolation periods, the low-pitched flat cluster 7 was the most common vocalization in the maternal potentiation test for both treatment groups. MAM exposure consistently reduced the use of the high-pitched short cluster 1 and increased the use of the low-pitched flat cluster 7, regardless of isolation (MAM treatment vs. controls for cluster 1 and cluster 7: *p* < 0.01 and *p* < 0.05, respectively; Fig. 9, Table S3).

There was no effect of sex on cluster profile.

*Cluster syntax* The cluster transition probability for males and females was pooled, as there were no significant main effects or interactions of sex regarding cluster distribution (see Table S3 in Supplementary Material 1).

**Fig. 10:**
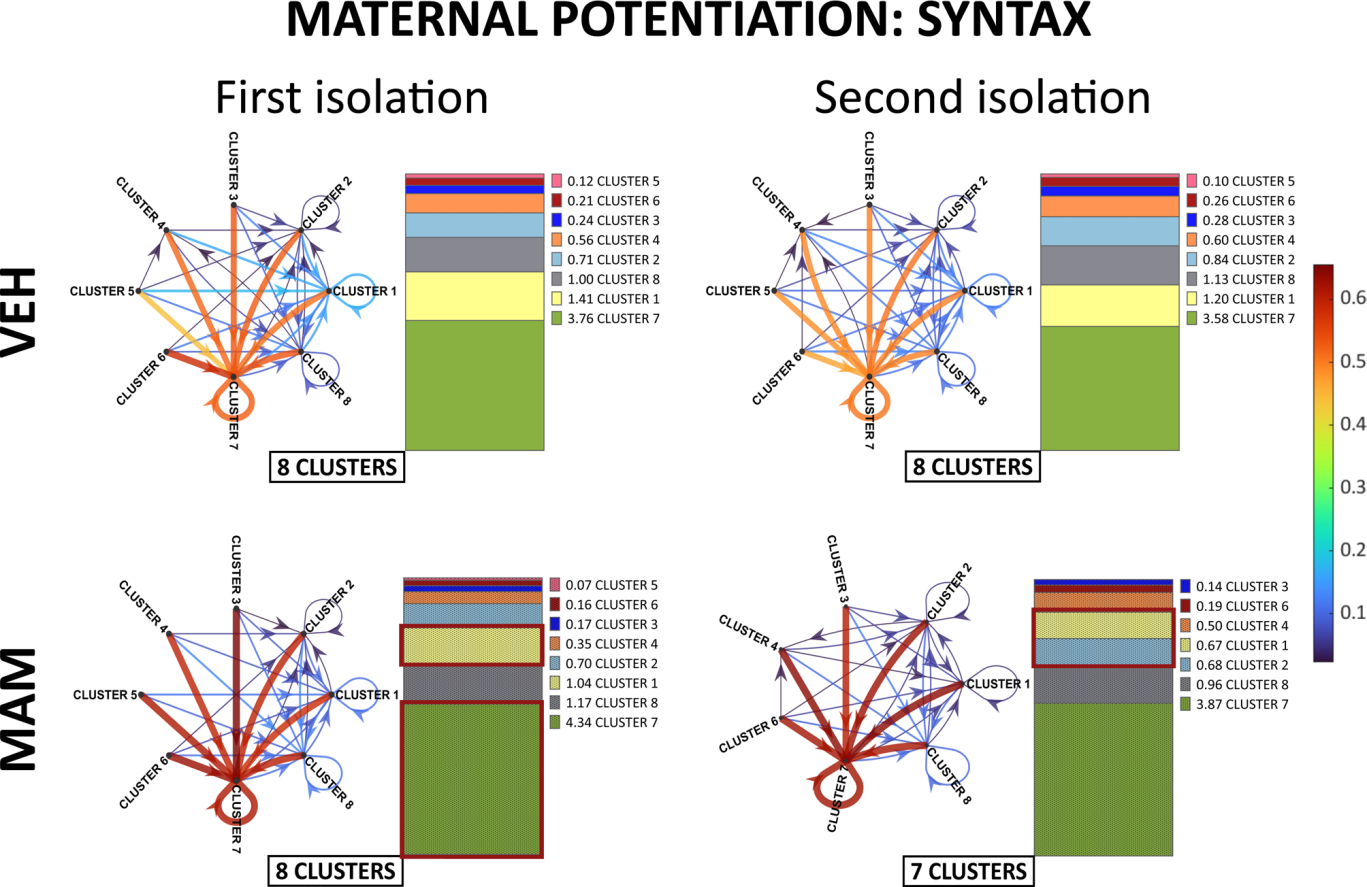
MAM treatment affects conditional probability for each cluster transition within USV bouts in the maternal potentiation test. The transitions between USV clusters in control and MAM-treated pups during the first isolation and the second isolation are illustrated in syntax flow paths representing the conditional probabilities of USVs transitioning from one call type to another. Arrows indicate the direction of transitions, with thicker arrows and brighter colors denoting higher transition probabilities. The vertical bar plots represent the summed conditional probabilities for cluster transitions within USV bouts in control and MAM-treated pups. These probabilities are sorted from the lowest to the highest within each treatment group and isolation period. In the first and second isolations, cluster 7 was the primary transition target. First isolation: control and MAM-treated pups transitioned between eight clusters. Compared to controls, MAM-treated pups were likelier to transition to low-pitch flat cluster 7 and less likely to transition to high-pitch short cluster 1. Second isolation: maternal contact affected the call syntax profile in MAM pups. Control pups continued transitioning between eight clusters, while MAM-treated pups transitioned between seven clusters. The syntax order changed in MAM pups: cluster 2, was third with the highest transition probability instead of cluster 1 that was in the first isolation.

During the first isolation, control and MAM-treated pups transitioned between eight clusters (Figs. 10, S6 in Supplementary Material 2). Cluster 7 had the highest probability of being the next cluster in the USV bout, including repeated use. Clusters 1 and 8 were the next most transitioned (Figs. 10, S6 in Supplementary Material 2). Compared to the control, MAM pups were more likely to transition to cluster 7 but less likely to transition to cluster 1 (Figs. 10, S6 in Supplementary Material 2).

Maternal contact did not affect the syntax of USV clusters in control pups but mildly influenced the syntax in MAM-treated pups. Firstly, MAM pups transitioned only between seven clusters. Secondly, the syntax order changed: cluster 2, not cluster 1 as in the first isolation, was third with the highest transition probability.

Overall, these analyses suggest that MAM treatment alters the distribution and syntax of USV call types, leading to a preference for low-pitched flat calls (cluster 7) over high-pitched short calls (cluster 1) and simplifying transition patterns, which may indicate impairments in vocal communication and social signaling. These results are consistent with the vocalization development experiment (Fig. 6).

## Discussion

The present study demonstrates that metylazoxymethanol acetate (MAM)-exposed rats exhibit compromised vocalization. As they age, MAM-exposed rats do not follow the typical changes observed in the controls, such as an increase in the number of USVs and a decrease in call duration. They consistently emit calls with lower frequencies throughout the developmental period (6th–12th PNDs). Moreover, the maternal potentiation effect, a proxy measure of maternal bond is absent or weakly expressed in MAM animals. The temporal arrangement of USVs was also sensitive to the toxin, as MAM-exposed pups started to vocalize later and demonstrated extended inter-call intervals. MAM rat calls are organized into fewer sequences with more bouts. Lastly, their vocal repertoire and syntax are less diverse, highlighting significant changes in the complexity and structure of their communication.

### The number of USVs

The development of rodent vocalizations demonstrates an increase in USVs from the earliest days of life, peaking at the 12th PND and followed by a gradual decline until weaning. This “trajectory” is associated with increasing independence from the mother, improved locomotor development, thermoregulation, and sensory maturation^24^. The ability to communicate at the earliest stages of development is crucial for further growth, and any disturbances in this process may impact social interactions in adulthood^32,63^.

In contrast to the control pups, the MAM-exposed animals exhibited fewer stress-induced calls. This reduction in USVs appears to be stable throughout the animal’s life, as we previously reported similar impairments in both juvenile and adult rats^26,53^. A reduced number of 40 kHz calls has also been observed in an immune model of schizophrenia involving LPS or Poly(I:C) exposure, as well as in neurexin and neuroligin knockout pups^45,46,73,74^. Deficits in vocalization in MAM pups align with human studies, where reduced speech quantity and quality (short, simple sentences) represents a negative symptom evident from infancy and persisting into adulthood in individuals with schizophrenia^16,75,76^.

Decreased number of isolation-induced calls may be due to a developmental delay. For instance, Stark et al.^55^ reported that prenatal MAM exposure delays neonatal reflexes at the early stages of rats’ life but not, as in the present study, nest-seeking behavior (Supplementary Material 3). While the body temperature of MAM-exposed animals was either higher or comparable to that of the controls (Figs. S1b, S1d in Supplementary Material 2), it did not correlate with the number of calls and, therefore, was not responsible for the communication deficit.

The lower number of USVs at the early stages of development and the lack of maternal potentiation might be due to a weakened bond with the mother^77^. According to Hofer et al.^34,78^, and Shair et al.^33^, pups learn to associate maternal retrieval with a reward (e.g., warmth, licking, grooming, feeding) during the reunion. Any disturbances in the maternal potentiation response could indicate learning and/or reward system complications. Thus, MAM pups may not perceive reunion with their mother as rewarding and, at the later stages of development, exhibit cognitive and social deficits, fewer ‘happy calls’, and impairments in the reward pathways^26,53,59,79,80^. Similarly, mice lacking the μ-opioid receptor gene, which mediates reward pathways, show deficits in maternal potentiation^81^.

The lower number of calls in MAM pups could also have been due to the impaired maternal behavior worsening the mother-infant relationship. However, in the present experiment, MAM-exposed dams retrieved their pups like control dams and showed comparable active care duration (Fig. S9 in Supplementary Material 2). One of the limitations of the present study was that we did not examine maternal responses to the pup’s calls.

The number of isolation-induced USVs serves also as a marker of the pups’ distress^82–84^. The reduced number of infant USVs may indicate disturbances in emotional expression due to stressful conditions. It has been demonstrated that MAM rats struggle to adapt their response to stress^56,57^. This phenomenon may be due to MAM-induced disturbances in brain regions responsible for socio-emotional regulation^80^.

### Basic USV features

Acoustic features of infant vocalizations, such as frequency and duration, encode the urgency of distress and influence caregiving behavior across species. In humans, caregivers interpret higher-pitched or prolonged cries as more urgent, prompting quicker and more intense responses^23,85^. Rodent dams show similar sensitivity, reacting more robustly to longer, lower-pitched USVs^86–88^. These parallels support the use of rodent vocalizations as a translational model for studying early communicative cues that shape maternal behavior in mammals.

In the present study, the control pups’ USVs had shortened with age, while MAM pups showed no change in call duration. Upon re-isolation, the MAM females emitted longer calls than the control females. With aging, the MAM offspring demonstrated an overall decrease in both the bandwidth and frequency of USVs agreeing with published reports^26,53^. Some studies using schizophrenia models found similar call disturbances, while others did not^45,46,73,74^.

The longer USV and flatter vocalizations, along with lower frequency in MAM pups, suggest disordered emotional expression, i.e., heightened anxiety and increased sensitivity to stress factors. Changes in the quality of basic USV features can predict adult emotional traits^89–91^. Indeed, in adulthood, MAM rats display increased anxiety-like behavior^57^. Compared to typical speech, intonation in schizophrenia patients shows reduced pitch variation^92–94^.

### Temporal arrangement

In addition to the basic acoustic features, the temporal organization of cries, such as inter-cry interval or speed of cries (call rate), is essential for increasing the perception of distress in human infants by caregivers^85,95^ and for eliciting maternal retrieval responses in rodent pups^28,87^. Variations in call organizations may indicate an early atypical affective state that could hinder the development of social communication.

The calls’ temporal arrangement differed between MAM and control pups. The time to initiate vocalization and inter-call interval were extended in MAM pups. We cannot rule out the possibility that the longer inter-call interval depends on the prolonged call duration. In contrast to our results, USVs of rats exposed to Poly(I:C) tended to occur closer together in time than those of the controls^54^. Notably, individuals with schizophrenia often display altered temporal features of speech, such as prolonged response times and increased frequency and duration of speech pauses, which negatively impact communication fluency^76,96,97^.

In the vocal development analysis, we observed fewer sequences in the MAM pups, and in the maternal potentiation experiment, the number of bouts was higher in MAM females than in the controls. The number of USVs per sequence and sequences per bout was reduced in MAM pups. Only control pups demonstrated fewer bouts at re-isolation compared to initial isolation and a higher number of sequences per bout. Disrupted temporal organization of USVs in MAM pups may reflect impairments in distress signaling, social communication, and the development of coordinated vocal expression.

### Cluster distribution and syntax

Recent research has identified two distinct categories of rodent *infant* USVs^83^. The first group includes calls with a frequency below 50 kHz, typically flat. The second group features higher-pitched and complex calls with frequencies above 50 kHz. As rat pups mature, low-pitched calls decrease, while higher-pitched calls become more prevalent^32,63^. Our study supports these observations because as the pups aged, the distribution of low-pitched flat clusters gradually decreased. At the same time, high-pitched short and “complex” clusters have increased. The maternal potentiation experiment revealed the differences in cluster distribution between treatment groups, not detected in the development experiment. Thus, MAM pups exhibited more low-pitched flat cluster and fewer high-pitched short calls cluster during the first and second isolation periods.

USV subtypes are not produced randomly but follow specific structures, grouped into phrases and motifs, forming complex communication patterns^29,31,98^. Our results revealed that MAM exposure simplified animals’ call transition profile with increased usage (~ 10%) of low-pitched, long, flat calls. Present data are the first showing that the sequencing of infant USVs is altered in the MAM model of schizophrenia. The cluster distribution and syntax results support our previous findings on the “flat pattern” in MAM rat’s vocalization^26,53^. The Poly(I:C) rats of schizophrenia likewise produce a lowered proportion of “complex” USVs and/or a higher proportion of flat calls^45,46^. The reduced vocal repertoire and simpler, fixed call sequences were also seen in other schizophrenia/ autism models, such as Poly(I:C) rats or TSC2 heterozygous mice^47,54,99^.

Low-pitched flat and high-pitched short infant calls are linked with negative valence^83,100–103^. While low-pitched calls are associated with stressful stimuli, high-pitched calls correspond to milder aversive states^100,104^. It has been proposed that the former might be an early version of the adult 22 kHz USV^83,100,104,105^. Indeed, we previously reported a higher amount of 22 kHz in MAM adolescent rats^23^. Thus, the higher proportion and more frequent transitions of low-pitched, flat clusters provide further evidence that MAM offspring exhibit heightened sensitivity to stress or impaired stress response.

### Sex effect

MAM affected the acoustic features of ultrasonic calls in both sexes almost equally. If anything, in female pups, the toxin produced a stronger disruptive effect on the number of USVs after potentiation, increased inter-call interval, and reduced the number of sequences per bout. This may be explained by males’ hyper-reactivity to separation from dams. Although some researchers postulate gender differences in schizophrenia, others dispute them^106–109^.

Regardless of the treatment, female pups consistently displayed shorter call durations and higher call frequency than males, possibly due to the greater stress resistance to maternal separation^26,110,111^. Bowers et al.^111^ found that male pups vocalized more and at a lower frequency than females, with dams more likely to retrieve male pups first. Alternatively, maternal factors, such as a known bias in maternal care, may play a role, as dams tend to groom male pups more often than females^112^. Also, in our study, dams retrieved male pups sooner during the reunion (Fig. S9 in Supplementary Material 2).

### Limitations and translational scope

While the MAM model captures several schizophrenia-relevant phenotypes, including disrupted vocal development, we acknowledge the limitations of directly applying our findings to human speech. Rodent USVs are non-verbal and emotionally driven signals that lack the semantic content and syntactic complexity of human language. Therefore, any parallels drawn must remain at the level of analogous patterns in vocal structure, such as call timing, bandwidth, frequency, and sequence organization, rather than functional or linguistic equivalence.

Moreover, our study focused exclusively on the vocal behavior of pups, without assessing maternal responses. This limits our insight into the bidirectional, socially mediated feedback loops that are essential for shaping early communication. Future research should incorporate dyadic interaction analyses and longitudinal follow-ups to better contextualize these vocal patterns within broader developmental trajectories.

Importantly, the implications of our findings extend beyond the search for the neurobiological basis of schizophrenia. The atypical development of vocalization observed in MAM-exposed pups may serve as an early behavioral marker of general neurodevelopmental disturbance^113^. This approach offers translational value for studying a range of conditions marked by early communicative impairments, such as autism spectrum disorders, developmental language delays, or early-life stress exposure^35,114^. By identifying conserved developmental patterns of disruption across species, this model can contribute to a more nuanced understanding of how early alterations in social communication emerge, evolve, and potentially inform early intervention strategies.

## Conclusion

Our results suggest that prenatal exposure to the metylazoxymethanol acetate toxin reduces ultrasonic vocalization in offspring. Since altered timing and sequencing deficits have been reported in schizophrenia patients, the rodent model based on ultrasonic call analysis may help in exploring their biological basis.

## Supplementary Information

Supplementary Information 1.

Supplementary Information 2.

Supplementary Information 3.

## Data Availability

The data supporting the findings of this study may be obtained from the authors upon request. For inquiries, please contact Agnieszka Potasiewicz at potasiew@if-pan.krakow.pl.
