# Supplementary material for: Infant rat ultrasonic vocalizations in the neurodevelopmental model of schizophrenia
Source: Sci Rep. 2025 Jul 28;15:27472. doi: 10.1038/s41598-025-08412-5 (PMC12304202; doi:10.1038/s41598-025-08412-5)
Supplement: Supplementary file 1 — Supplementary Information 1. [file 41598_2025_8412_MOESM1_ESM.docx]

Infant rat ultrasonic vocalizations in the neurodevelopmental model of schizophrenia

Supplementary material

Agnieszka Potasiewicz^1^*, Zuzanna Mincikiewicz^1^,Piotr Popik^1^, Agnieszka Nikiforuk^1^

Department of Behavioral Neuroscience and Drug Development, Maj Institute of Pharmacology, Polish Academy of Sciences, Krakow, Poland

Corresponding author*:

Agnieszka Potasiewicz,
Maj Institute of Pharmacology, Polish Academy of Sciences,
12 Smetna Street, 31-343 Krakow, Poland,
e-mail: [potasiew@if-pan.krakow.pl](mailto:potasiew@if-pan.krakow.pl)
Tel: +4812 6623374; Fax: +4812 6374500

For identification on subsequent experimental days, the pups were marked using colored, odorless markers (**Table S1**) according to the following pattern: individuals numbered 1-4 were marked in black, numbers 5-8 in green, numbers 9-12 in blue, and numbers 13-16 in red. Starting from the first animal, every fourth one had its left front paw marked. Every fourth rat, starting from the second one, had its right front paw marked. Every fourth animal starting from the third one had its left hind paw marked, and every fourth animal starting from the fourth one had its right hind paw marked.

**Table S1** Color and paw marking patterns for identification of experimental rats.

| A type of paw  Color | Front  left | Front  right | Hind  left | Hind  right |
| --- | --- | --- | --- | --- |
| Black | Rat 1 | Rat 2 | Rat 3 | Rat 4 |
| Green | Rat 5 | Rat 6 | Rat 7 | Rat 8 |
| Blue | Rat 9 | Rat 10 | Rat 11 | Rat 12 |
| Red | Rat 13 | Rat 14 | Rat 15 | Rat 16 |

**Table S2** Statistical comparisons of USV parameters in the vocal development experiment.

| Call characteristic | Source of variation | F [DFn, DFd] / H / Chi^2^ [DFn, N] | p Value |
| --- | --- | --- | --- |
| Number of USVs | Treatment | F [1,116] = 5.88 | **0.0169** |
|  | Sex | F [1,116] = 0 | 0.9839 |
|  | Treatment × Sex | F [1,116] = 0 | 0.9964 |
|  | PND | F [1.8, 209.8] = 17.28 | **0** |
|  | PND × Treatment | F [1.8, 209.8] = 3.63 | **0.0324** |
|  | PND × Sex | F [1.8, 209.8] = 1.03 | 0.3535 |
|  | PND × Treatment × Sex | F [1.8, 209.8] = 0.67 | 0.4977 |
|  |  |  |  |
| Number of USVs per minute: 6^th^PND | Treatment | F [1, 116] = 0.33 | 0.5693 |
|  | Sex | F [1, 116] = 0.05 | 0.8305 |
|  | Treatment × Sex | F [1, 116] = 0.04 | 0.8354 |
|  | Time | F [3.4, 396.6] = 8.33 | **0** |
|  | Time × Treatment | F [3.4, 396.6] = 2.49 | **0.0517** |
|  | Time× Sex | F [3.4, 396.6] = 1.17 | 0.3239 |
|  | Time × Treatment × Sex | F [3.4, 396.6] = 0.12 | 0.9629 |
|  |  |  |  |
| Number of USVs per minute: 9^th^ PND | Treatment | F [1, 116] = 0.82 | 0.3668 |
|  | Sex | F [1, 116] = 0.96 | 0.3283 |
|  | Treatment × Sex | F [1, 116] = 0.44 | 0.5093 |
|  | Time | F [2, 234.8] = 13.84 | **0** |
|  | Time × Treatment | F [2, 234.8] = 1.61 | 0.2018 |
|  | Time × Sex | F [2, 234.8] = 4.47 | **0.0121** |
|  | Time × Treatment × Sex | F [2, 234.8] = 0.84 | 0.4324 |
|  |  |  |  |
| Number of USVs per minute: 12^th^ PND | Treatment | F [1, 116] = 8.26 | **0.0048** |
|  | Sex | F [1, 116] = 0.43 | 0.5125 |
|  | Treatment × Sex | F [1, 116] = 0.42 | 0.5259 |
|  | Time | F [2.6, 307] = 28.35 | **0** |
|  | Time × Treatment | F [2.6, 307] = 0.28 | 0.817 |
|  | Time × Sex | F [2.6, 307] = 0.78 | 0.4939 |
|  | Time × Treatment × Sex | F [2.6, 307] = 0.42 | 0.7139 |
|  |  |  |  |
| Mean USV duration | Treatment | F [1,116] = 0.6 | 0.4393 |
|  | Sex | F [1,116] = 6.24 | **0.0139** |
|  | Treatment × Sex | F [1,116] = 0.04 | 0.8366 |
|  | PND | F [2, 232] = 2.16 | 0.1171 |
|  | PND × Treatment | F [2, 232] = 5.01 | **0.0074** |
|  | PND × Sex | F [2, 232] = 0.74 | 0.4759 |
|  | PND × Treatment × Sex | F [2, 232] = 1.73 | 0.1795 |
|  |  |  |  |
| Bandwidth | Treatment | F [1,116] = 0.32 | 0.5702 |
|  | Sex | F [1,116] = 0.56 | 0.4552 |
|  | Treatment × Sex | F [1,116] = 0.20 | 0.6558 |
|  | PND | F [1.9, 200] = 109.03 | **0** |
|  | PND × Treatment | F [1.9, 200] = 0.81 | 0.4396 |
|  | PND × Sex | F [1.9, 200] = 1.01 | 0.3629 |
|  | PND × Treatment × Sex | F [1.9, 200] = 0.04 | 0.9585 |
|  |  |  |  |
| Peak frequency | Treatment | F [1,116] = 5.19 | **0.0246** |
|  | Sex | F [1,116] = 4.57 | **0.0346** |
|  | Treatment × Sex | F [1,116] = 0 | 0.9989 |
|  | PND | F [1.5, 176] = 15.18 | **0** |
|  | PND × Treatment | F [1.5, 176] = 0.24 | 0.7246 |
|  | PND × Sex | F [1.5, 176] = 2.56 | 0.0946 |
|  | PND × Treatment × Sex | F [1.5, 176] = 0.15 | 0.8036 |
|  |  |  |  |
| Latency to the first USV | PND (Friedman ANOVA) | Chi Sqr. [2, N = 106] = 1.26 | 0.5315 |
| Fourteen outliers were excluded from the analysis: four control females, two control males, four MAM females, and four MAM males | 6^th^ PND (Kruskal-Wallis ANOVA by Ranks) | H [3, N = 106] = 1.86 | 0.6423 |
|  | 9^th^ PND (Kruskal-Wallis ANOVA by Ranks) | H [3, N = 106] = 8.56 | **0.0357** |
|  | 12^th^ PND (Kruskal-Wallis ANOVA by Ranks) | H [3, N= 106] = 4.8 | 0.1869 |
|  |  |  |  |
| Inter-call interval | Treatment | F [1, 109] = 15.69 | **0.0001** |
| Seven outliers were excluded from the analysis: two control females, two control males, and three MAM females | Sex | F [1, 109] = 0.01 | 0.9349 |
|  | Treatment × Sex | F [1, 109] = 0.14 | 0.7087 |
|  | PND | F [2, 218] = 0.36 | 0.6964 |
|  | PND × Treatment | F [2, 218] = 1.13 | 0.3248 |
|  | PND × Sex | F [2, 218] = 0.40 | 0.6713 |
|  | PND × Treatment × Sex | F [2, 218] = 0.14 | 0.8705 |
|  |  |  |  |
| Number of sequences | Treatment | F [1, 116] = 3.98 | **0.0483** |
|  | Sex | F [1, 116] = 0.61 | 0.4367 |
|  | Treatment × Sex | F [1, 116] = 0.62 | 0.4335 |
|  | PND | F [1.8, 212.6] = 25.8 | **0** |
|  | PND × Treatment | F [1, 116] = 0.56 | 0.5599 |
|  | PND × Sex | F [1, 116] = 0.8 | 0.44 |
|  | PND × Treatment × Sex | F [1, 116] = 1.59 | 0.2081 |
|  |  |  |  |
| Number of USVs per sequence | Treatment | F [1, 116] = 4.49 | **0.0363** |
|  | Sex | F [1, 116] = 0.22 | 0.6366 |
|  | Treatment × Sex | F [1, 116] = 1.12 | 0.2917 |
|  | PND | F [1.5, 177.4] = 26.22 | **0** |
|  | PND × Treatment | F [1.5, 177.4] = 2.04 | 0.1449 |
|  | PND × Sex | F [1.5, 177.4] = 0.02 | 0.9627 |
|  | PND × Treatment × Sex | F [1.5, 177.4] = 0.11 | 0.8387 |
|  |  |  |  |
| Number of bouts | Treatment | F [1, 116] = 1.85 | 0.1768 |
|  | Sex | F [1, 116] = 0.04 | 0.8485 |
|  | Treatment × Sex | F [1, 116] = 0.10 | 0.7465 |
|  | PND | F [1.7, 199.2] = 24.87 | **0** |
|  | PND × Treatment | F [1.7, 199.2] = 2.28 | 0.1132 |
|  | PND × Sex | F [1.7, 199.2] = 2.49 | **0.094** |
|  | PND × Treatment × Sex | F [1.7, 199.2] = 0.49 | 0.5839 |
|  |  |  |  |
| Number of sequences per bout | Treatment | F [1, 116] = 5.24 | **0.0238** |
|  | Sex | F [1, 116] = 0.54 | 0.4625 |
|  | Treatment × Sex | F [1, 116] = 1.89 | 0.1721 |
|  | PND | F [1.1, 126] = 14.59 | **0.0001** |
|  | PND × Treatment | F [1.1, 126] = 6.14 | **0.0126** |
|  | PND × Sex | F [1.1, 126] = 2.63 | 0.1048 |
|  | PND × Treatment × Sex | F [1.1, 126] = 1.57 | 0.2142 |
|  |  |  |  |
| Call type distribution | Treatment | F [1, 116] = 3.16 | **0.0783** |
|  | Sex | F [1, 116] = 0.55 | 0.4618 |
|  | Treatment × Sex | F [1, 116] = 1.57 | 0.2129 |
|  | PND | F [1.8, 212] = 44.74 | **0** |
|  | PND × Treatment | F [1.8, 212] = 0.30 | 0.7217 |
|  | PND × Sex | F [1.8, 212] = 1.54 | 0.2188 |
|  | PND × Treatment × Sex | F [1.8, 212] = 0.26 | 0.7498 |
|  | Cluster | F [1.9, 222.7] = 517.19 | **0** |
|  | Cluster × Treatment | F [1.9, 222.7] = 0.53 | 0.5793 |
|  | Cluster × Sex | F [1.9, 222.7] = 0.73 | 0.4758 |
|  | Cluster × Treatment × Sex | F [1.9, 222.7] = 0.72 | 0.4838 |
|  | PND × Cluster | F [4, 468] = 101.28 | **0** |
|  | PND × Cluster × Treatment | F [4, 468] = 0.65 | 0.6294 |
|  | PND × Cluster × Sex | F [4, 468] = 1.07 | 0.3693 |
|  | PND × Cluster × Treatment × Sex | F [4, 468] = 0.43 | 0.7873 |
|  |  |  |  |
| Body weight | Treatment | F [1, 116] = 34.18 | **0** |
|  | Sex | F [1, 116] = 8.38 | **0.0045** |
|  | Treatment × Sex | F [1, 116] = 10.08 | 0.7825 |
|  | PND | F [1.4, 159.7] = 1776.56 | **0** |
|  | PND × Treatment | F [1.4, 159.7] = 32.67 | **0** |
|  | PND × Sex | F [1.4, 159.7] = 3.09 | **0.0671** |
|  | PND × Treatment × Sex | F [1.4, 159.7] = 0.21 | 0.722 |
|  |  |  |  |
| Body temperature | Treatment | F [1, 116] = 19.11 | **0** |
|  | Sex | F [1, 116] = 0.05 | 0.8227 |
|  | Treatment × Sex | F [1, 116] = 0.56 | 0.4562 |
|  | PND | F [1.7, 193.9] = 1776.56 | **0.0016** |
|  | PND × Treatment | F [1.7, 193.9] = 32.67 | 0.3336 |
|  | PND × Sex | F [1.7, 193.9] = 3.09 | 0.5406 |
|  | PND × Treatment × Sex | F [1.7, 193.9] = 0.21 | 0.0664 |
|  |  |  |  |
| Litter effect on the number of USV | Treatment | F [1, 100] = 10.1 | **0.002** |
|  | Sex | F [1, 100] = 0.28 | 0.6005 |
|  | Treatment × Sex | F [1, 100] = 2.7 | 0.1034 |
|  | Litter (nested in Treatment) | F [8, 100] = 10.49 | **0** |
|  | Sex × Litter (nested in Treatment) | F [8, 100] = 1.08 | 0.384 |
|  | PND | F [2, 16] = 17.37 | **0** |
|  | PND × Treatment | F [2, 16] = 4.29 | **0.015** |
|  | PND × Sex | F [2, 16] = 1.74 | 0.1776 |
|  | PND × Treatment × Sex | F [2, 16] = 0.44 | 0.647 |
|  | PND × Litter (nested in Treatment) | F [2, 16] = 4.79 | **0** |
|  | **PND × Sex × Litter (nested in Treatment)** | **F [2, 16] = 0.89** | **0.5801** |
|  |  |  |  |

**Table S3** Statistical comparisons of USV parameters in the maternal potentiation experiment.

| Call characteristic | Source of variation | F (DFn, DFd) | p Value |
| --- | --- | --- | --- |
| Number of USVs | Treatment | F [1,113] = 8.92 | **0.0035** |
|  | Sex | F [1,113] = 0 | 0.9974 |
|  | Treatment × Sex | F [1,113] = 6.57 | **0.0117** |
|  | Isolation | F [1,113] = 29.01 | **0** |
|  | Isolation × Treatment | F [1,113] = 1.28 | 0.2611 |
|  | Isolation × Sex | F [1,113] = 0 | 0.9516 |
|  | Isolation × Treatment × Sex | F [1,113] = 1.04 | 0.3098 |
|  |  |  |  |
| Number of USVs per minute:  FIRST ISOLATION | Treatment | F [1, 113] = 4.63 | **0.0336** |
|  | Sex | F [1, 113] = 0 | 0.9674 |
|  | Treatment × Sex | F [1, 113] = 3.29 | 0.0725 |
|  | Time | F [2.8, 319] = 9.13 | **0** |
|  | Time × Treatment | F [2.8, 319] = 2.06 | 0.1088 |
|  | Time × Sex | F [2.8, 319] = 1.32 | 0.2676 |
|  | Time × Treatment × Sex | F [2.8, 319] = 0.43 | 0.7171 |
|  |  |  |  |
| Number of USVs per minute:  SECOND ISOLATION | Treatment | F [1, 113] = 7.91 | **0.0058** |
|  | Sex | F [1, 113] = 0 | 0.9781 |
|  | Treatment × Sex | F [1, 113] = 5.94 | **0.0163** |
|  | Time | F [2.4, 267.3] = 24.68 | **0** |
|  | Time × Treatment | F [2.4, 267.3] = 0.91 | 0.4167 |
|  | Time × Sex | F [2.4, 267.3] = 1.16 | 0.3274 |
|  | Time × Treatment × Sex | F [2.4, 267.3] = 1.17 | 0.3155 |
|  |  |  |  |
| Mean USV duration | Treatment | F [1,113] = 3.51 | 0.0636 |
|  | Sex | F [1,113] = 3.47 | 0.0653 |
|  | Treatment × Sex | F [1,113] = 2.27 | 0.1348 |
|  | Isolation | F [1,113] = 121.26 | **0** |
|  | Isolation × Treatment | F [1,113] = 2.17 | 0.1436 |
|  | Isolation × Sex | F [1,113] = 4.43 | **0.0374** |
|  | Isolation × Treatment × Sex | F [1,113] = 6.53 | 0**.0119** |
|  |  |  |  |
| Bandwidth | Treatment | F [1,113] = 4.32 | **0.0399** |
|  | Sex | F [1,113] = 1.21 | 0.2744 |
|  | Treatment × Sex | F [1,113] = 0.20 | 0.6534 |
|  | Isolation | F [1,113] = 8.79 | **0.0037** |
|  | Isolation × Treatment | F [1,113] = 0.37 | 0.5462 |
|  | Isolation × Sex | F [1,113] = 0.89 | 0.3484 |
|  | Isolation × Treatment × Sex | F [1,113] = 0.02 | 0.8925 |
|  |  |  |  |
| Peak frequency | Treatment | F [1,113] = 14.75 | **0.0002** |
|  | Sex | F [1,113] = 0.71 | 0.4002 |
|  | Treatment × Sex | F [1,113] = 2.57 | 0.1118 |
|  | Isolation | F [1,113] = 2.37 | 0.1265 |
|  | Isolation × Treatment | F [1,113] = 0.71 | 0.4008 |
|  | Isolation × Sex | F [1,113] = 4.64 | **0.0333** |
|  | Isolation × Treatment × Sex | F [1,113] = 0.02 | 0.2593 |
|  |  |  |  |
| Latency to the first USV | Treatment | F [1, 107] = 0.37 | 0.5457 |
| Six outliers were excluded from the analysis: one control female, three control males, and two MAM males | Sex | F [1, 107] = 3.87 | 0.0517 |
|  | Treatment × Sex | F [1, 107] = 1.58 | 0.2109 |
|  | Isolation | F [1, 107] = 0.58 | 0.4493 |
|  | Isolation × Treatment | F [1, 107] = 6.47 | **0.0124** |
|  | Isolation × Sex | F [1, 107] = 0.63 | 0.4282 |
|  | Isolation × Treatment × Sex | F [1, 107] = 0.92 | 0.3388 |
|  |  |  |  |
| Inter-call interval | Treatment | F [1, 105] = 3.93 | 0.05 |
| Eight outliers were excluded from the analysis: two control females, two control males, two MAM females, and two MAM males | Sex | F [1, 105] = 1.11 | 0.2945 |
|  | Treatment × Sex | F [1, 105] = 8.12 | **0.0053** |
|  | Isolation | F [1, 105] = 27.45 | **0** |
|  | Isolation × Treatment | F [1, 105] = 0.05 | 0.8256 |
|  | Isolation × Sex | F [1, 105] = 0.98 | 0.3253 |
|  | Isolation × Treatment × Sex | F [1, 105] = 0.04 | 0.8368 |
|  |  |  |  |
| Number of sequences | Treatment | F [1, 113] = 2.06 | 0.1543 |
|  | Sex | F [1, 113] = 0.53 | 0.4688 |
|  | Treatment × Sex | F [1, 113] = 1.69 | 0.1959 |
|  | Isolation | F [1, 113] = 0.01 | 0.9353 |
|  | Isolation × Treatment | F [1, 113] = 0.12 | 0.7267 |
|  | Isolation × Sex | F [1, 113] = 0.03 | 0.8675 |
|  | Isolation × Treatment × Sex | F [1, 113] = 0 | 0.9740 |
|  |  |  |  |
| Number of USVs per sequence | Treatment | F [1, 113] = 5.52 | **0.0205** |
|  | Sex | F [1, 113] = 0.39 | 0.5316 |
|  | Treatment × Sex | F [1, 113] = 3.87 | 0.0515 |
|  | Isolation | F [1, 113] = 52.49 | **0** |
|  | Isolation × Treatment | F [1, 113] = 1.39 | 0.2412 |
|  | Isolation × Sex | F [1, 113] = 0.19 | 0.6618 |
|  | Isolation × Treatment × Sex | F [1, 113] = 1.16 | 0.2844 |
|  |  |  |  |
| Number of bouts | Treatment | F [1, 113] = 8.56 | **0.0041** |
|  | Sex | F [1, 113] = 0.40 | 0.5279 |
|  | Treatment × Sex | F [1, 113] = 7.74 | **0.0063** |
|  | Isolation | F [1, 113] = 12.99 | **0.0005** |
|  | Isolation × Treatment | F [1, 113] = 1.11 | 0.2933 |
|  | Isolation × Sex | F [1, 113] = 0.23 | 0.6302 |
|  | Isolation × Treatment × Sex | F [1, 113] = 0.29 | 0.5886 |
|  |  |  |  |
| Number of sequences per bout | Treatment | F [1, 109] = 14.32 | **0.0003** |
| Four outliers were excluded from the analysis: one control female, one control male, one MAM female, and one MAM male | Sex | F [1, 109] = 0.65 | 0.4214 |
|  | Treatment × Sex | F [1, 109] = 6.67 | **0.0111** |
|  | Isolation | F [1, 109] = 16.68 | **0.0001** |
|  | Isolation × Treatment | F [1, 109] = 9.93 | **0.0021** |
|  | Isolation × Sex | F [1, 109] = 2.46 | 0.1198 |
|  | Isolation × Treatment × Sex | F [1, 109] = 4.56 | **0.035** |
|  |  |  |  |
| Call type distribution | Treatment | F [1, 113] = 8.20 | **0.005** |
|  | Sex | F [1, 113] = 0.02 | 0.9017 |
|  | Treatment × Sex | F [1, 113] = 2.45 | 0.1204 |
|  | Isolation | F [1, 113] = 4.16 | **0.0438** |
|  | Isolation × Treatment | F [1, 113] = 6 | **0.0158** |
|  | Isolation × Sex | F [1, 113] = 2.86 | 0.0937 |
|  | Isolation × Treatment × Sex | F [1, 113] = 2.39 | 0.1248 |
|  | Cluster | F [1.5, 170.6] = 299.64 | **0** |
|  | Cluster × Treatment | F [1.5, 170.6] = 4.70 | **0.0178** |
|  | Cluster × Sex | F [1.5, 170.6] = 0.89 | 0.3870 |
|  | Cluster × Treatment × Sex | F [1.5, 170.6] = 1.27 | 0.2773 |
|  | Isolation × Cluster | F [2.4, 273] = 4.54 | **0.0073** |
|  | Isolation × Cluster × Treatment | F [2.4, 273] = 1.14 | 0.3261 |
|  | Isolation × Cluster × Sex | F [2.4, 273] = 2.01 | 0.1263 |
|  | Isolation × Cluster × Treatment × Sex | F [2.4, 273] = 1.69 | 0.1799 |
|  |  |  |  |
| Active maternal care | Treatment | F [1, 94] = 2.2 | 0.1434 |
|  | Sex | F [1, 94] = 2.8 | 0.0959 |
|  | Treatment × Sex | F [1, 94] = 0.6 | 0.4236 |
|  |  |  |  |
| Latency to 1^st^ approach to pup | Treatment | F [1, 91] = 0.29 | 0.5897 |
| Three outliers were excluded from the analysis: one control male, one MAM female, and one MAM male | Sex | F [1, 91] = 5.17 | **0.0254** |
|  | Treatment × Sex | F [1, 91] = 0.01 | 0.9163 |
|  |  |  |  |
| Body weight | Treatment | F [1, 113] = 29.23 | **0** |
|  | Sex | F [1, 113] = 0.93 | 0.3377 |
|  | Treatment × Sex | F [1, 113] = 0.14 | 0.7128 |
|  |  |  |  |
| Body temperature | Treatment | F [1, 113] = 0.08 | 0.7722 |
|  | Sex | F [1, 113] = 0.69 | 0.4086 |
|  | Treatment × Sex | F [1, 113] = 0.12 | 0.7349 |
|  | Isolation | F [1, 113] = 34.59 | **0** |
|  | Isolation × Treatment | F [1, 113] = 0 | 0.9996 |
|  | Isolation × Sex | F [1, 113] = 1.91 | 0.1692 |
|  | Isolation × Treatment × Sex | F [1, 113] = 0.24 | 0.6256 |
|  |  |  |  |
| Litter effect on the number of USV | Treatment | F [1, 97] = 8.03 | **0.0056** |
|  | Sex | F [1, 97] = 0.49 | 0.4843 |
|  | Treatment × Sex | F [1, 97] = 3.96 | **0.0495** |
|  | Litter (nested in Treatment) | F [1, 97] = 5.97 | **0** |
|  | Sex × Litter (nested in Treatment) | F [1, 97] = 0.96 | 0.4713 |
|  | Isolation | F [1, 97] = 31.38 | **0** |
|  | Isolation × Treatment | F [1, 97] = 2.03 | 0.1573 |
|  | Isolation × Sex | F [1, 97] = 1.05 | 0.308 |
|  | Isolation × Treatment × Sex | F [1, 97] = 0.01 | 0.9124 |
|  | Isolation × Litter (nested in Treatment) | F [8, 97] = 6.17 | **0** |
|  | **Isolation × Sex × Litter (nested in Treatment)** | **F [8, 97] = 1.16** | **0.33** |
|  |  |  |  |

**Table S4** Characteristics of vocalization clusters identified in the vocal development experiment.

| **Collapsed clusters** | **VEA clusters** | **Peak Freq (kHz)** | | | **Call Length (s)** | | | **Delta Freq (kHz)** | | | **Slope (kHz/s)** | | |
| --- | --- | --- | --- | --- | --- | --- | --- | --- | --- | --- | --- | --- | --- |
|  |  | PND6 | PND9 | PND12 | PND6 | PND9 | PND12 | PND6 | PND9 | PND12 | PND6 | PND9 | PND12 |
| **Cluster 1** | Cluster 2 | 64.6 | 64.9 | 64.3 | 0.01 | 0.01 | 0.02 | 5.7 | 4.4 | 6.5 | 581 | 341 | 449 |
|  | Cluster 10 | 75.9 | 73.8 | 73.2 | 0.01 | 0.01 | 0.02 | 4.2 | 5.8 | 7.4 | 398 | 389 | 444 |
|  | Cluster 15 | 58.1 | 61.2 | 59.9 | 0.01 | 0.01 | 0.01 | 3.0 | 2.3 | 2.8 | 81 | 38 | 35 |
|  | Cluster 20 | 64.6 | 65.4 | 64.3 | 0.02 | 0.02 | 0.02 | 5.6 | 3.9 | 4.5 | -28 | 99 | 82 |
| **Cluster 2** | Cluster 14 | 43.3 | 40.3 | 39.7 | 0.12 | 0.14 | 0.14 | 12.8 | 17.8 | 17.0 | -4 | -41 | -26 |
|  | Cluster 12 | 44.5 | 42.3 | 41.6 | 0.09 | 0.09 | 0.08 | 19.6 | 24.0 | 23.4 | -146 | -208 | -204 |
| **Cluster 3** | Cluster 19 | 52.2 | 51.2 | 49.8 | 0.06 | 0.04 | 0.04 | 19.9 | 24.9 | 25.3 | -707 | -696 | -601 |
| **Cluster 4** | Cluster 1 | 43.5 | 42.3 | 40.7 | 0.14 | 0.14 | 0.15 | 10.7 | 16.2 | 15.2 | 3 | -26 | -17 |
|  | Cluster 4 | 47.6 | 46.7 | 45.4 | 0.09 | 0.07 | 0.06 | 15.4 | 22.3 | 21.9 | -96 | -178 | -190 |
| **Cluster 5** | Cluster 6 | 50.2 | 49.2 | 48.8 | 0.08 | 0.03 | 0.04 | 13.0 | 24.2 | 27.1 | 187 | 530 | 473 |
| **Cluster 6** | Cluster 8 | 44.4 | 44.6 | 42.2 | 0.15 | 0.16 | 0.15 | 7.2 | 9.7 | 9.6 | 16 | 16 | 18 |
| **Cluster 7** | Cluster 3 | 47.7 | 45.4 | 44.1 | 0.13 | 0.13 | 0.12 | 6.5 | 5.9 | 6.0 | 2 | 8 | 2 |
|  | Cluster 7 | 42.3 | 41.1 | 39.7 | 0.10 | 0.12 | 0.11 | 4.3 | 4.8 | 5.2 | 26 | 29 | 35 |
|  | Cluster 9 | 42.7 | 40.1 | 39.3 | 0.09 | 0.11 | 0.11 | 3.6 | 3.5 | 3.6 | -3 | -4 | -5 |
|  | Cluster 11 | 44.1 | 41.8 | 39.9 | 0.13 | 0.14 | 0.12 | 6.0 | 5.8 | 5.8 | 53 | 45 | 81 |
|  | Cluster 13 | 43.5 | 41.2 | 40.6 | 0.10 | 0.11 | 0.08 | 6.1 | 6.3 | 7.7 | 66 | 74 | 124 |
|  | Cluster 16 | 42.0 | 41.4 | 39.7 | 0.10 | 0.12 | 0.12 | 3.1 | 4.4 | 4.5 | 19 | 18 | 14 |
|  | Cluster 17 | 45.8 | 44.4 | 42.4 | 0.09 | 0.09 | 0.08 | 5.7 | 6.0 | 5.6 | 33 | 51 | 69 |
|  | Cluster 21 | 40.4 | 39.8 | 38.9 | 0.10 | 0.13 | 0.15 | 2.3 | 3.8 | 4.2 | -6 | -8 | -5 |
|  | Cluster 23 | 40.5 | 38.1 | 37.3 | 0.08 | 0.09 | 0.11 | 2.4 | 3.5 | 4.2 | -27 | -36 | -35 |
| **Cluster 8** | Cluster 5 | 44.7 | 42.4 | 41.2 | 0.09 | 0.08 | 0.08 | 5.2 | 4.3 | 4.0 | -47 | -44 | -44 |
|  | Cluster 18 | 47.5 | 46.7 | 44.9 | 0.02 | 0.02 | 0.02 | 3.9 | 3.4 | 4.2 | -52 | 51 | 17 |
|  | Cluster 22 | 41.6 | 39.6 | 38.8 | 0.02 | 0.02 | 0.01 | 1.3 | 1.5 | 2.2 | -101 | -50 | -23 |

Values represent the average measurements across all experimental groups (control and MAM-exposed males and females).

**Table S5** Summary of findings: vocal development and maternal potentiation experiments.

| Parameter | Vocalization development | | | Maternal Potentiation (MP) | | |
| --- | --- | --- | --- | --- | --- | --- |
|  | **VEH** | **MAM** | **Figure** | **VEH** | **MAM** | **Figure** |
| Number of USVs | Increased with age (M, F) | Stable across postnatal days  Lower than control at 12th PND (M, F) | 2A-B | MP present ˗ more USVs after maternal contact (M, F) | MP weak (M) or absent (F) | 8A-B |
| Duration | Decreased with age (M, F) | Stable across postnatal days (M, F) | 2C | Increased after maternal contact (M, F) | Increased after maternal contact (M, F)  Longer compared to controls (F) | 8C |
| Bandwidth | Increased with age (M, F) | Increased with age (M, F) | 2D | Lack of changes after reunion (M, F) | Increased after reunion (F)  Flatter compared to controls (M, F) | 8D |
| Peak frequency | Increased with age (M, F) | Lower than control (M, F) | 2E | Lack of changes after reunion (M, F) | Decreased after reunion (F)  Lower than controls (M, F) | 8E |
| Latency to the first USV | Stable across days (M, F) | Delayed at than control 9th PND (F) | 3B | Stable across isolations periods (M, F) | Shorter after maternal contact (F) | 9A |
| Inter-Call Interval | Stable across days (M, F) | Stable across days (M, F)  Longer than control (M, F) | 3C | Lower after maternal contact (M, F) | Stable across isolations periods (M)  Increased compared to controls (F) | 9B |
| Number of USV sequences | Varied across days (M, F) | Varied across days (M, F)  Lower than control | 3D | Stable across isolations periods (M, F) | Stable across isolations periods (M, F) | 9C-D |
| USV per sequence | Increased with age (M, F) | Increased with age (M, F)  Lower than control (M, F) | 3E | Increased after maternal contact (M, F) | Increased after maternal contact (M, F)  Lower than control (M, F) | 9C-D |
| Number of USV bouts | Decreased with age (M, F) | Decreased with age (M, F) | 3F | Decreased after maternal contact (M, F) | Stable across isolations periods (M, F)  Increased compared to controls (F) | 9E |
| Sequences per bout | Increased with age (M, F) | Stable across postnatal days (M, F)  Lower than control at 12th PND (M, F) | 3G | Increased after maternal contact (F) | Stable across isolations periods (M, F)  Decreased compared to controls (F) | 9F |
| Cluster distribution | High-pitched calls increased with age (M, F) | Comparable to control (M, F) | 5 | Low-pitched flat calls increased, the high-pitched calls decreased during the re-isolation (M, F) | Low-pitched flat calls increased, the high-pitched calls decreased during the re-isolation (M, F)  Compared to controls, reduced high-pitched calls and increased flat, low-pitched calls (M, F) | 10 |
| Call syntax | More varied with age (M, F) | More varied with age (M, F),  Simplified at 9th PND (more transitions to flat calls, M, F) | 6 | Stable across isolations periods (M, F) | Simplified (more transitions to flat calls, M, F) in comparison to controls | 11 |

Abbreviations: VEH - vehicle control, MAM - methylazoxymethanol acetate, F - female, M - male, PND - postnatal day
