# Supplementary material for: Infant rat ultrasonic vocalizations in the neurodevelopmental model of schizophrenia
Source: Sci Rep. 2025 Jul 28;15:27472. doi: 10.1038/s41598-025-08412-5 (PMC12304202; doi:10.1038/s41598-025-08412-5)
Supplement: Supplementary file 2 — Supplementary Information 2. [file 41598_2025_8412_MOESM2_ESM.docx]

Infant rat ultrasonic vocalizations in the neurodevelopmental model of schizophrenia

Supplementary material

Agnieszka Potasiewicz^1^*, Zuzanna Mincikiewicz^1^, Piotr Popik^1^, Agnieszka Nikiforuk^1^

Department of Behavioral Neuroscience and Drug Development, Maj Institute of Pharmacology, Polish Academy of Sciences, Krakow, Poland

Corresponding author*:

Agnieszka Potasiewicz,
Maj Institute of Pharmacology, Polish Academy of Sciences,
12 Smetna Street, 31-343 Krakow, Poland,
e-mail: [potasiew@if-pan.krakow.pl](mailto:potasiew@if-pan.krakow.pl)
Tel: +4812 6623374; Fax: +4812 6374500


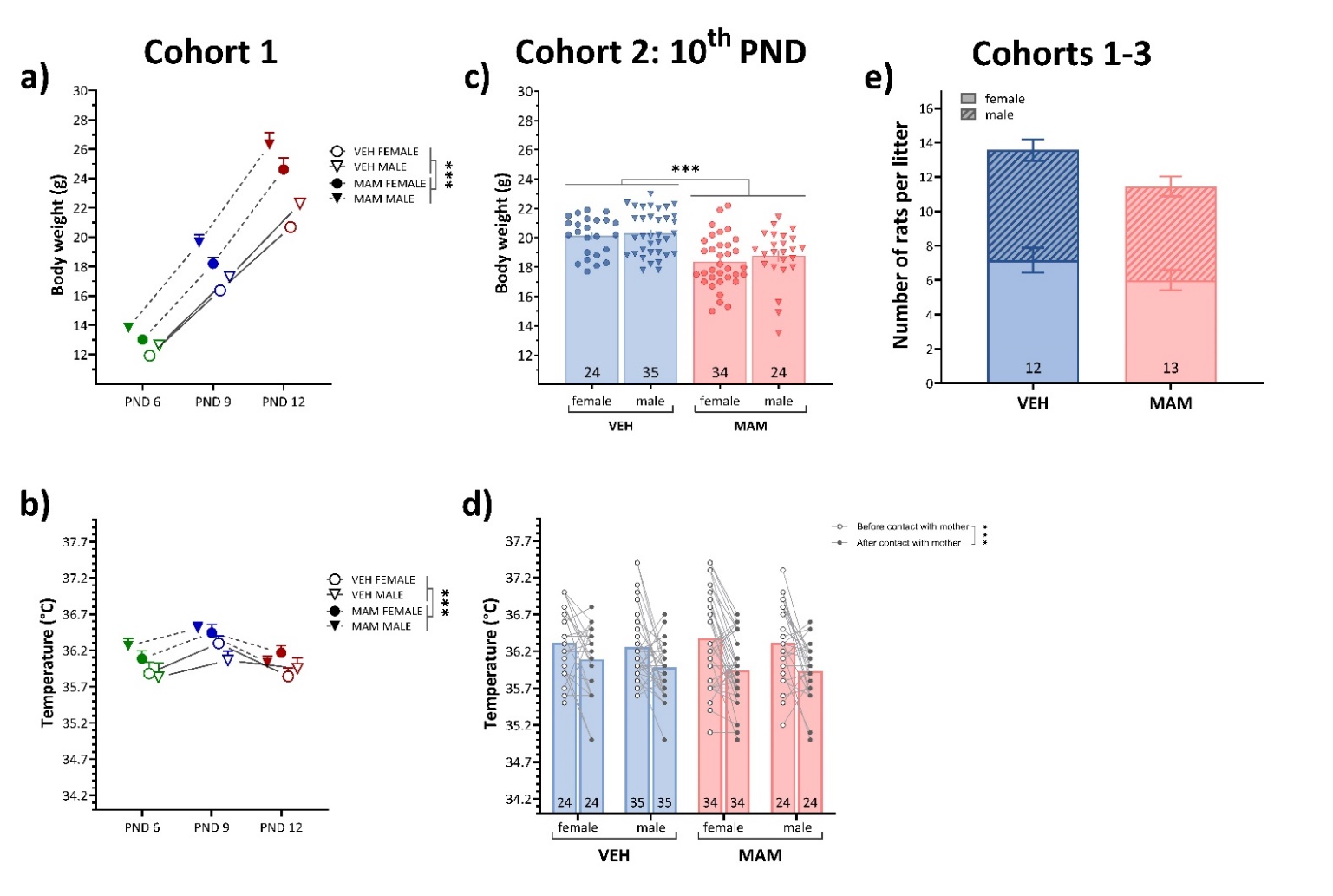


**Figure S1 Litter characteristics and physiological parameters of control and MAM-treated pups across postnatal development.** **(b)** The body weight of pups in cohort 1 measured on the 6th, 9th, and 12th postnatal days (PND). MAM-treated pups weighed more than controls across all time points. **(a)** The body temperature of pups in cohort 1 measured on the 6th, 9th, and 12th postnatal days. MAM-treated pups had higher body temperatures than controls across all time points. **(c)** The body weight of pups in cohort 2 measured during the maternal potentiation test (10th postnatal day). MAM-treated pups weighed less than controls. **(d)** The body temperature of pups in cohort 2 measured during the maternal potentiation test (10th postnatal day). MAM-treated pups had body temperature similar to that of the control. **(e)** The average number of rats per litter across cohorts 1–3 (cohort 1 – USV development experiment, cohort 2 - the maternal potentiation test, cohort 3 – home-seeking behavior – see Supplementary Material 3). Bar segments indicate the female-to-male ratio within each group. The average number of pups in control and MAM-treated litters was similar. The number of pups in control and MAM-treated litters was similar. Points represent the mean ± S.E.M. Statistical significance is indicated as follows: **p < 0.01, ***p < 0.001. The annotations at the bottom of the bar indicate the sample size.


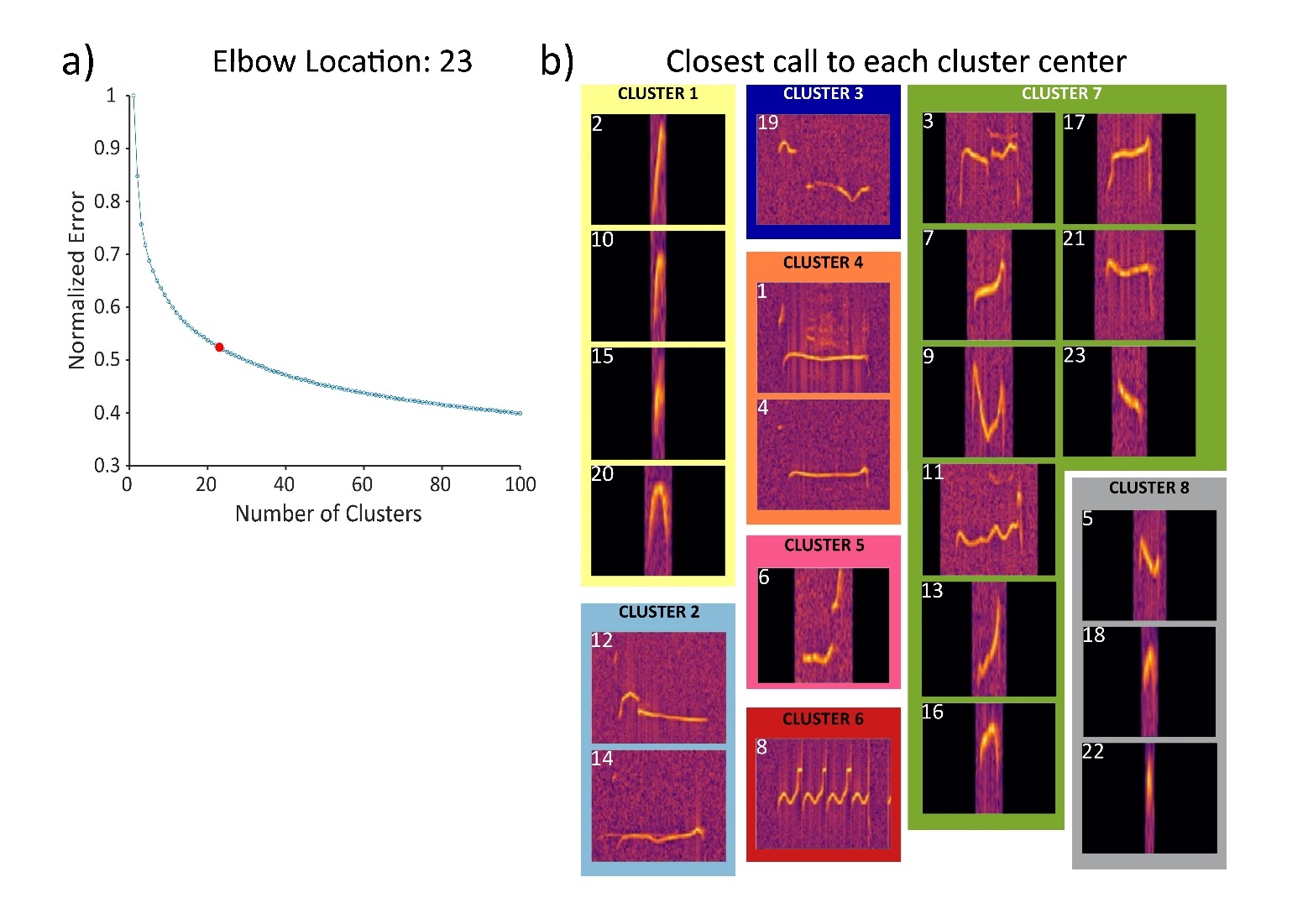


**Figure S2 Clustering analysis of USVs using Variational Autoencoder (VAE).** Clustering was performed using an unsupervised VAE model, with elbow optimization conducted through the DeepSqueak software. The VAE model was trained on 212,196 USVs recorded across 6th postnatal day (60,305 USVs), 9th postnatal day (69,055 USVs), and 12th postnatal day (82,836 USVs) from both sexes (females and males), and treatment groups (control and MAM). **(a)** An elbow plot was used to determine the optimal number of clusters for USV calls. The normalized error decreased as the number of clusters increased, with the "elbow" point identified at 23 clusters (red dot). This point indicates where additional clusters provide diminishing improvements in the error metric. **(b)** Spectrograms of the closest calls to each cluster center demonstrate the diversity of USV calls captured during clustering. The VAE model identified 23 clusters, which were collapsed into eight larger clusters based on cluster characteristics and the UMAP projection.


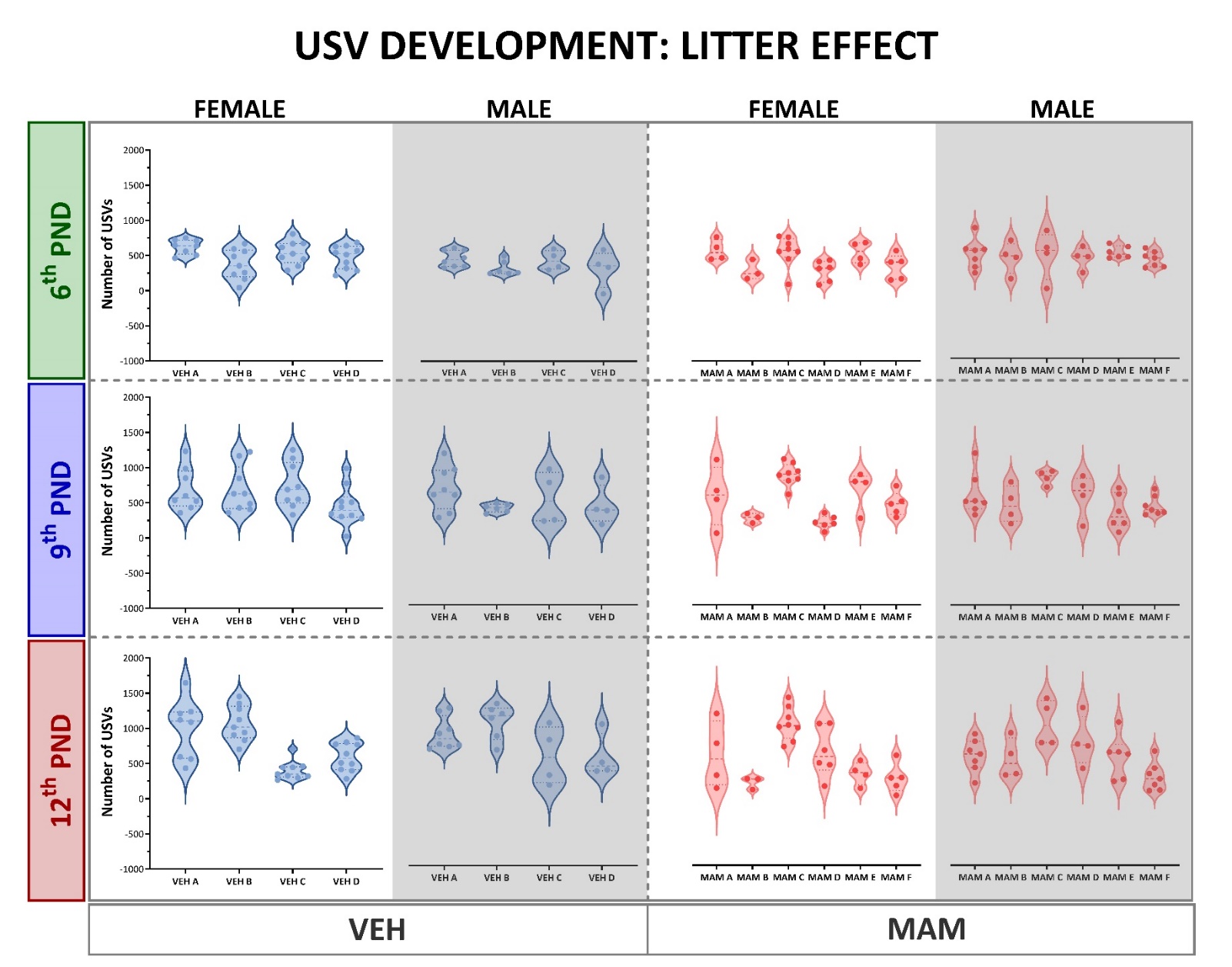


**Figure S3 The litter had no effect on the number of USVs during postnatal development in both control and MAM-treated pups.** Violin plots display the number of USVs produced by individual litters of control (VEH) and MAM-treated female and male pups on the 6th, 9th, and 12th postnatal days. Each violin corresponds to a specific litter (VEH: A–D, MAM: A–F). Each dot represents a single pup. Data are separated by sex and litter to illustrate within-litter variability. USV production was consistent across VEH and MAM litters, with no significant litter-to-litter differences observed.


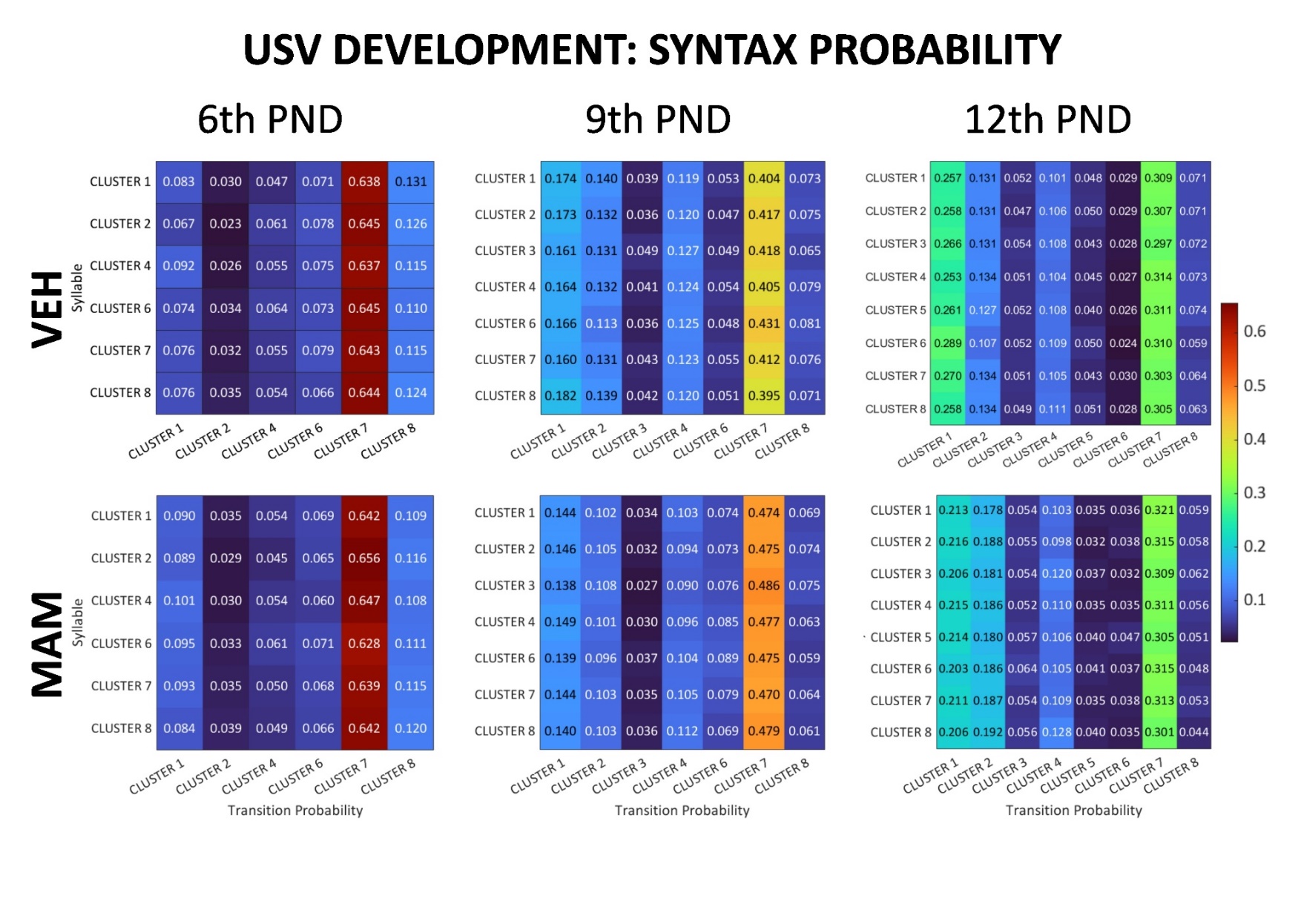


**Figure S4 Syntax analysis of USV clusters across postnatal development in control and MAM-treated pups.** Heatmaps represent the transition probabilities between USV clusters (Clusters 1–8) within bouts for control (VEH) and MAM-treated pups on the 6^th^, 9^th^, and 12^th^ postnatal days. Each cell indicates the likelihood of transitioning from one cluster (row) to another (column), with probabilities ranging from 0 to 0.6 (color-coded, as shown by the scale bar).


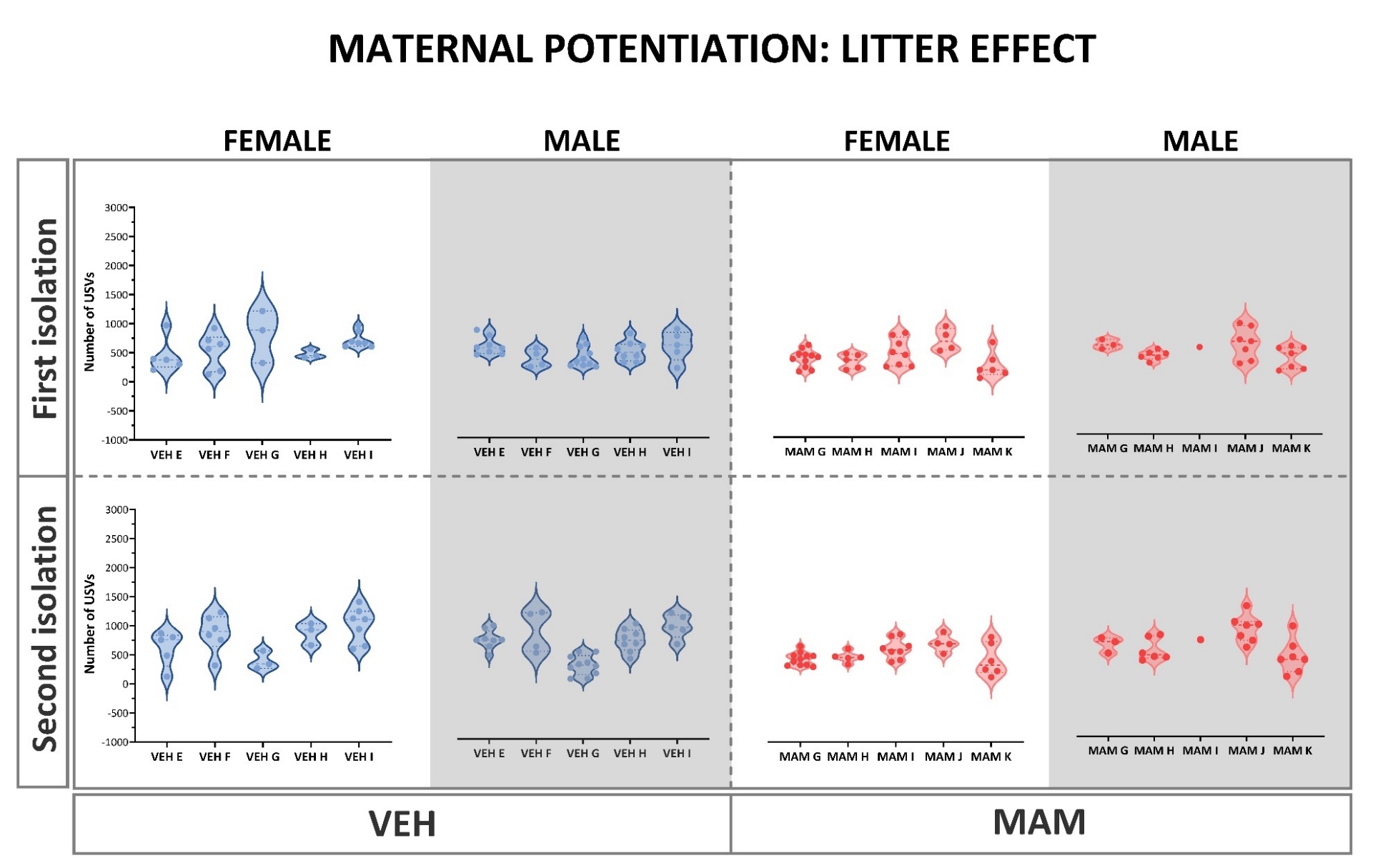


**Figure S5 The litter had no effect on the number of USVs in the maternal potentiation tests.** Violin plots show the number of USVs produced by individual litters of control (VEH) and MAM-treated female and male pups during the first isolation (before maternal contact) and the second isolation (after maternal contact). Each violin represents data from a specific litter (VEH: E–I, MAM: G–K). Each dot represents a single pup. Data are separated by sex and litter to illustrate within-litter variability. The number of USVs did not vary across litters during the first and second isolation periods.


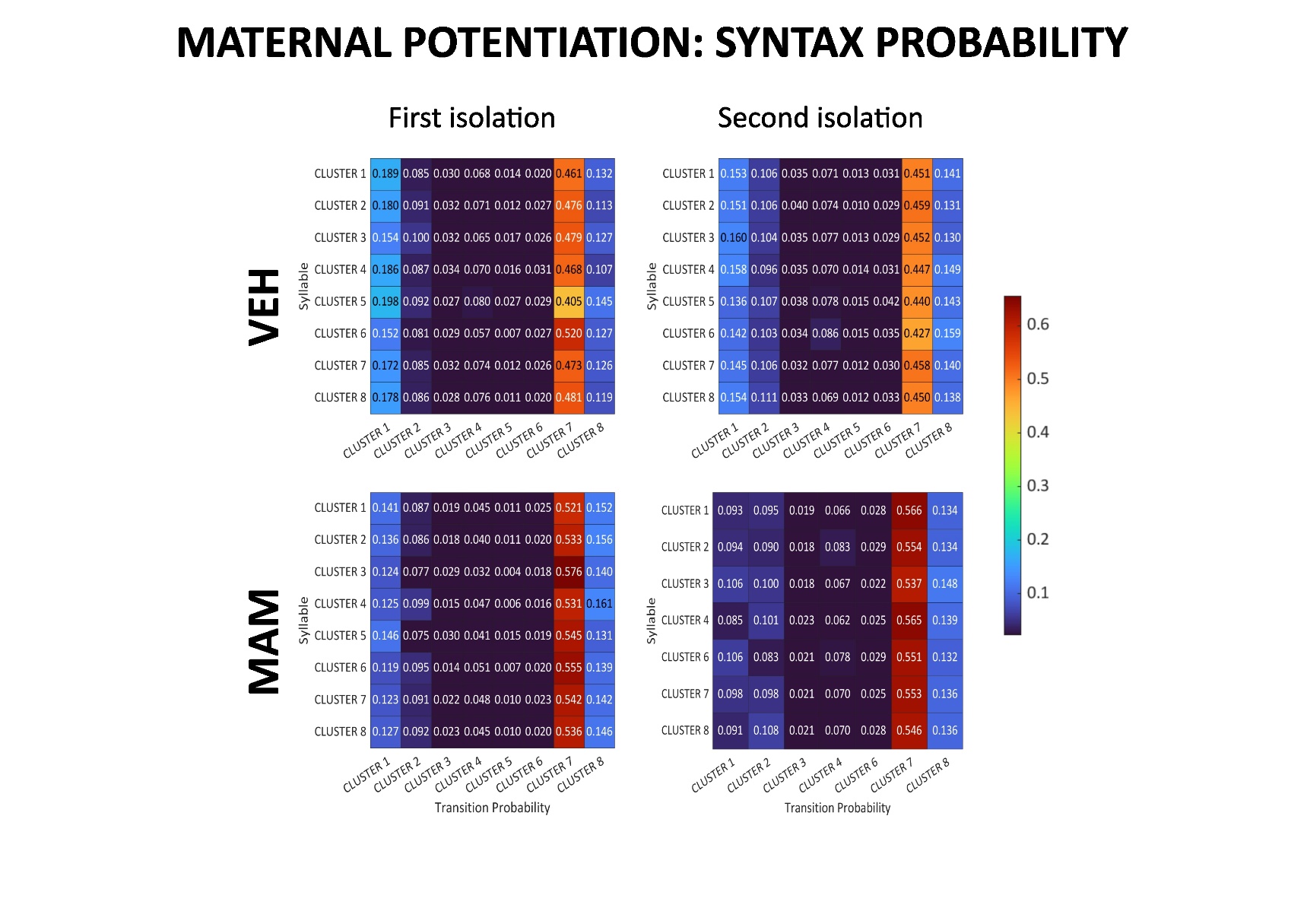


**Figure S6 Syntax analysis of USV clusters across postnatal development in control and MAM-treated pups.** Heatmaps represent the transition probabilities between USV clusters (Clusters 1–8) within bouts for control (VEH) and MAM-treated pups on the 6^th^, 9^th^, and 12^th^ postnatal days. Each cell indicates the likelihood of transitioning from one cluster (row) to another (column), with probabilities ranging from 0 to 0.6 (color-coded, as shown by the scale bar).


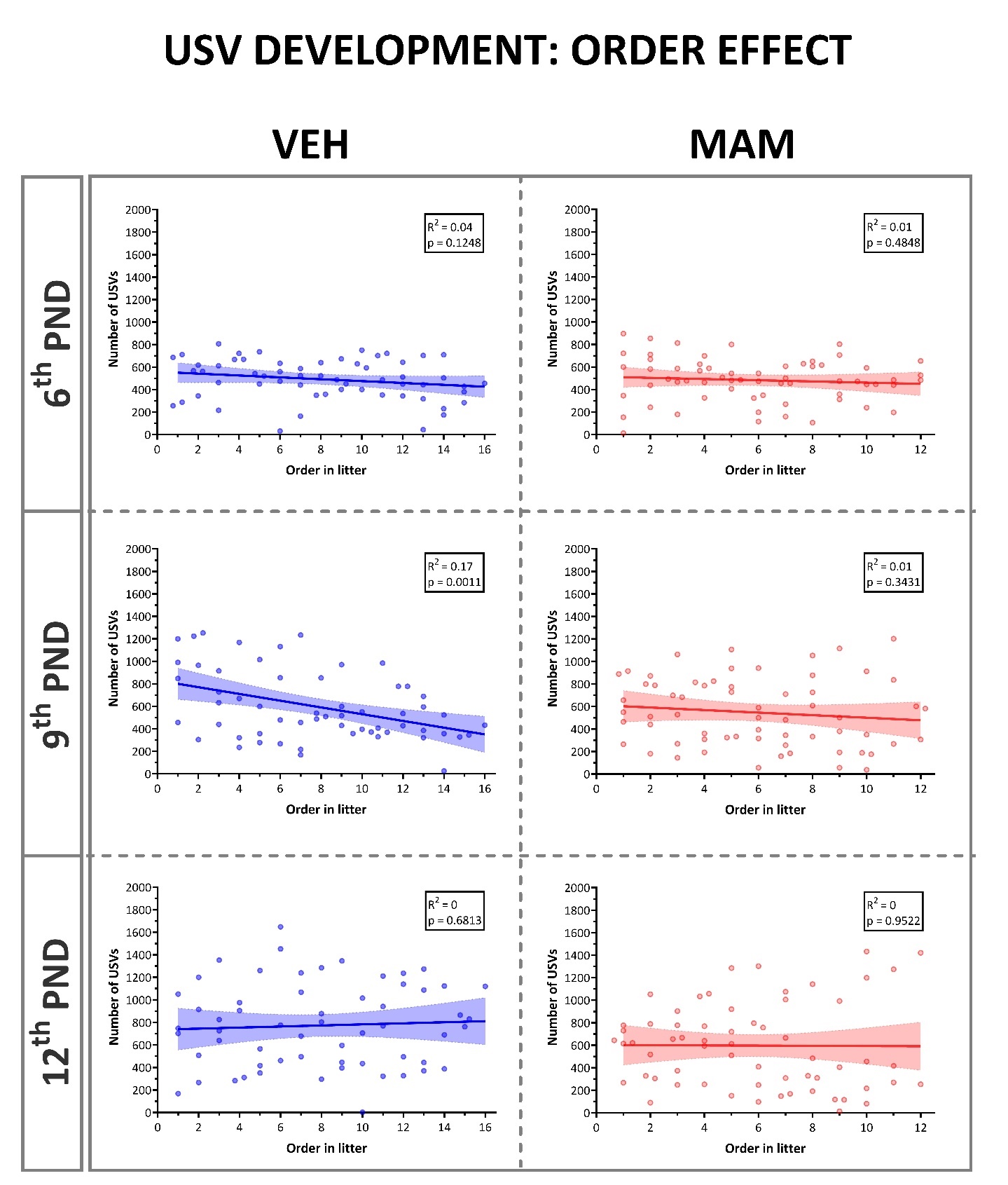


**Figure S7 Testing order does not influence USV emission during development.** Scatter plots with linear regression lines showing the relationship between testing order within a litter and the total number of USVs on the 6th, 9th, and 12th postnatal days (PND) in control (VEH) and MAM-exposed pups (male and female). A weak negative association was observed only in VEH pups on 9^th^ PND (R² = 0.17, p = 0.0011); no significant effects were found at other time points or in the MAM group. These data support the conclusion that testing order had no consistent or systematic impact on vocalization output during development.


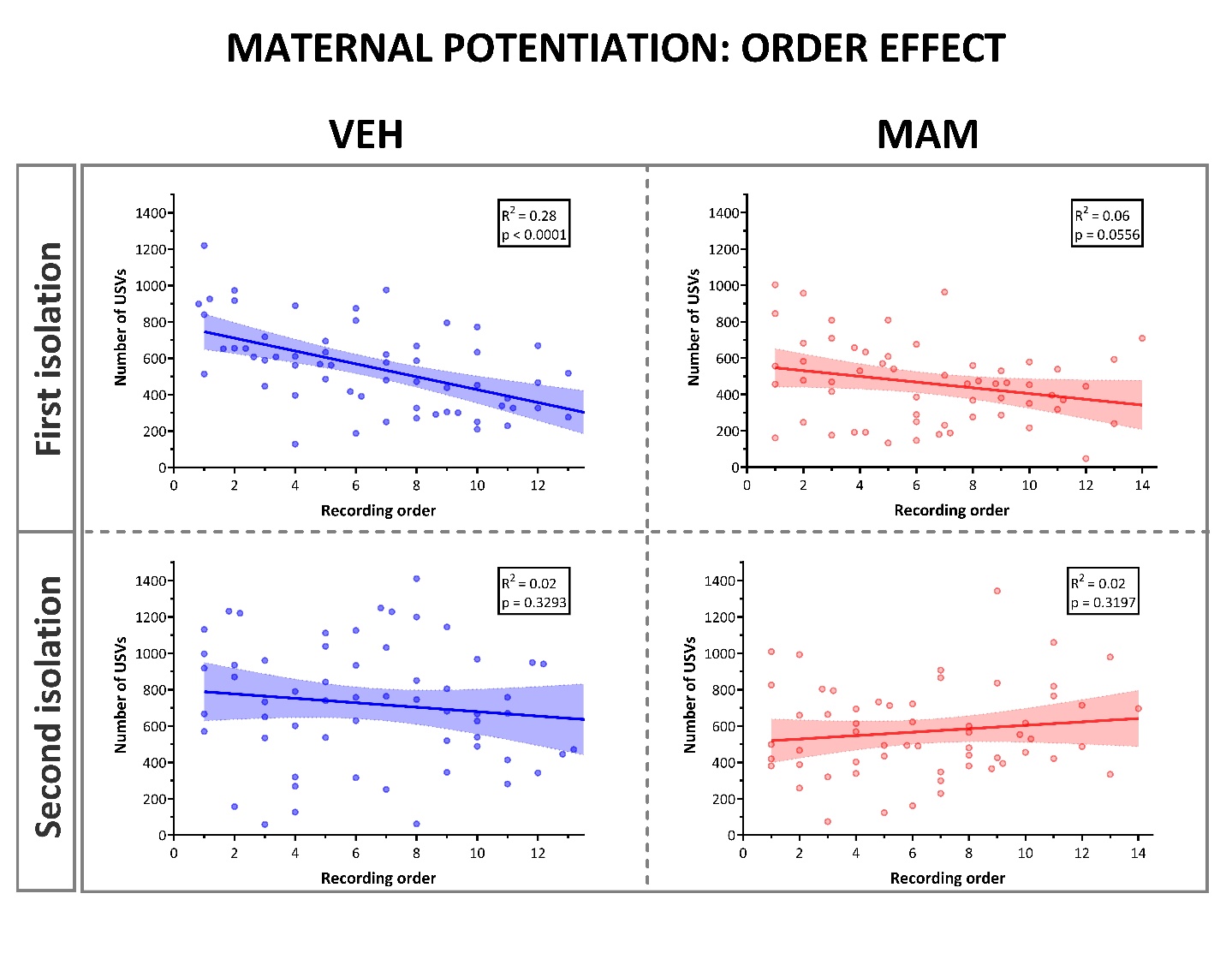


**Figure S8 Testing order does not influence USV emission in the Maternal Potentiation Test.** Linear regression plots showing the relationship between recording order within a litter and the number of USVs emitted during the first and second isolation phases in control (VEH) and MAM-exposed pups (male and female). A modest order effect was observed only in VEH pups during the first isolation (R² = 0.22, p < 0.0001), with no effects in the MAM group or during the second isolation. These findings indicate that testing order does not represent a confounding variable in the maternal potentiation outcomes.

**Maternal behavior was not affected by interactions with MAM-treated offspring**

18 dam-pup recordings (out of a total of 117) were excluded from the analysis due to video or technical issues.

Prenatal MAM exposure did not affect dams' time spent on active care for their offspring (**Figure S9a**). Similarly, the latency to the first approach to a pup was comparable between the MAM and control groups (**Figure S9b**). Notably, the latency to the first approach by the dam was shorter for the male than female pups, an effect observed for both treated groups (the main effect of the sex: F[1, 91] = 15.17, p < 0.05; **Figure S9b**).

**
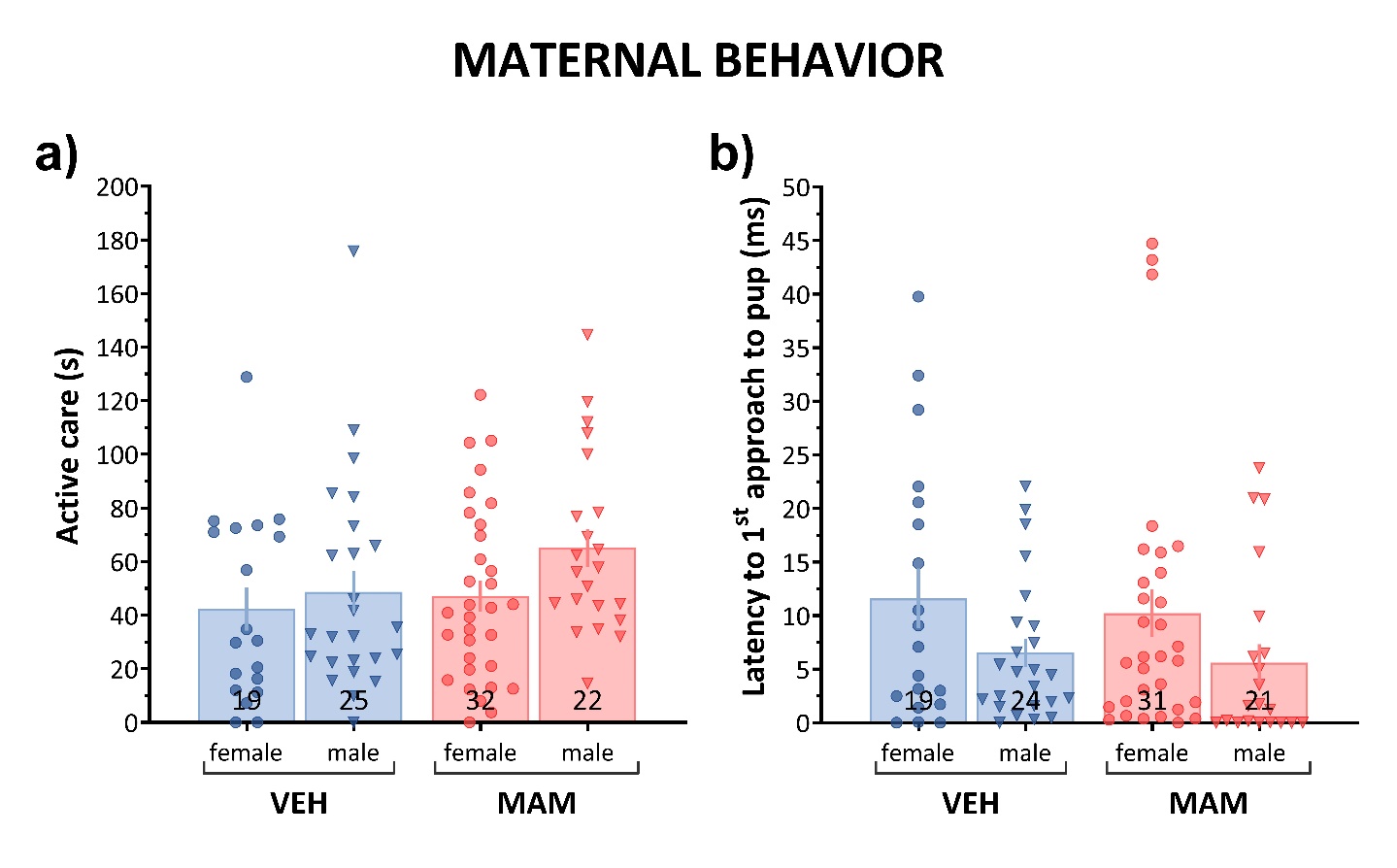
**

**Figure S9 Maternal behavior toward control and MAM-treated pups during reunion in the maternal potentiation test. (a)** Time spent on active care toward and MAM-treated pups by dams. Active care includes licking, grooming, and retrieval behaviors. No significant differences in care were observed between the dams of control and MAM offspring. **(b)** The latency to the first approach toward pup did not differ between dams of control and MAM offspring. Dams approached male pups sooner than female pups. Points represent the mean ± S.E.M. The annotations at the bottom of the bar indicate the sample size.
