# Supplementary material for: Infant rat ultrasonic vocalizations in the neurodevelopmental model of schizophrenia
Source: Sci Rep. 2025 Jul 28;15:27472. doi: 10.1038/s41598-025-08412-5 (PMC12304202; doi:10.1038/s41598-025-08412-5)
Supplement: Supplementary file 3 — Supplementary Information 3. [file 41598_2025_8412_MOESM3_ESM.docx]

Infant rat ultrasonic vocalizations in the neurodevelopmental model of schizophrenia

Supplementary material

Agnieszka Potasiewicz^1^*, Zuzanna Mincikiewicz^1^ , Piotr Popik^1^, Agnieszka Nikiforuk^1^

Department of Behavioral Neuroscience and Drug Development, Maj Institute of Pharmacology, Polish Academy of Sciences, Krakow, Poland

Corresponding author*:

Agnieszka Potasiewicz,

Maj Institute of Pharmacology, Polish Academy of Sciences,

12 Smetna Street, 31-343 Krakow, Poland,

e-mail: [potasiew@if-pan.krakow.pl](mailto:potasiew@if-pan.krakow.pl)

Tel: +4812 6623374; Fax: +4812 6374500


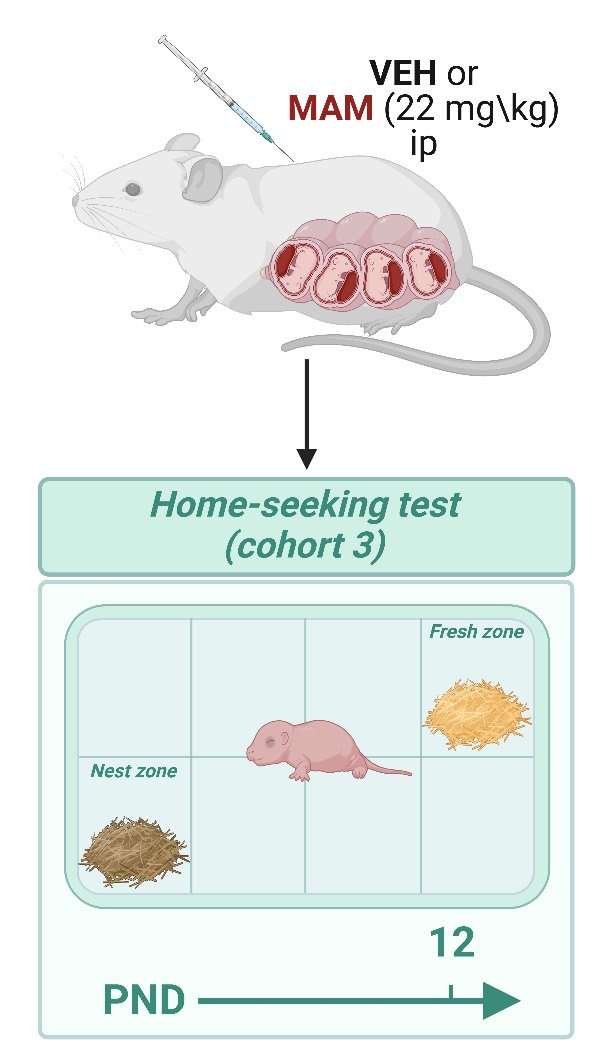
***Nest-seeking test (12^th^ PND) – cohort 3***

**Figure S7 The procedure of the nest-seeking test.** VEH – vehicle, MAM - methylazoxymethanol acetate, PND – postnatal day.

**Procedure:**

The nest-seeking test was conducted on 64 rat pups from three VEH control litters (J–K) and two MAM-treated litters (L–M), with detailed information provided in **Table S6**.

The testing procedure was adapted from a study by (Scattoni et al., 2005; Tonkiss et al., 1996). The testing box consisted of a rectangular, black polycarbonate apparatus (43 × 30 × 6 cm) that was virtually divided into eight equal zones, as illustrated in **Figure S7**. Two opposite zones (counterbalanced) contained bedding from the pup's home cage or an equal amount of fresh, novel bedding. The bedding was spread under the mesh floor. On the 12^th^ PND, each pup was gently placed in the exact center of the apparatus, counterbalancing the orientation between animals. The pup was recorded for 3 minutes while the following measures were analyzed: latency to reach the nest zone, the number of entries to the nest zone, time spent in the nest zone, and total distance traveled. If the pup failed to enter either the home cage bedding or the fresh bedding sector, a maximum latency of 180 seconds was scored. The camera above the arena recorded the pup's behavior, which was measured automatically by the Any-maze® tracking system (Stoelting Co., Illinois, USA). Each pup was tested only once.

**Table S6 Number of rats assessed in the nest-seeking test.** Treatment: prenatal methylazoxymethanol acid (MAM) or vehicle (VEH).

| **Cohort** | **Procedure** |  | **VEH**  **female** | **VEH**  **male** | **MAM**  **female** | **MAM**  **male** |
| --- | --- | --- | --- | --- | --- | --- |
| The third cohort | The nest-seeking test | Number of rats | 23 | 19 | 8 | 15 |
|  |  | Number of litters | 3 (J–L) | | 2 (L–M) | |
|  |  | Average litter size | 15 |  | 12 |  |

**Results:**

**Table S7** illustrates similar nest-seeking behaviors between prenatally MAM-treated and control female and male pups. Both groups reached the nest zone in a comparable time, made a similar number of entries into the nest zone, and spent similar durations within it. Additionally, the MAM-treated pups traveled distances comparable to those of the control group. For statistics, see **Table S8.**

**Table S7 Behavioral responses during the nest-seeking test.**

|  | **VEH**  **female** | | |  | **VEH**  **male** | | | | **MAM**  **female** | | |  | **MAM**  **male** | | |
| --- | --- | --- | --- | --- | --- | --- | --- | --- | --- | --- | --- | --- | --- | --- | --- |
| Latency to the first nest zone entries (s) | 123.9 | ± | 12.8 |  | 108.4 | ± | 14.7 |  | 99.7 | ± | 21.1 |  | 105.9 | ± | 15 |
| Number of entries to nest zone | 2.4 | ± | 0.6 |  | 3.7 | ± | 0.8 |  | 4 | ± | 1.3 |  | 3.3 | ± | 0.9 |
| Time spent in the nest zone (s) | 51 | ± | 12.3 |  | 54.7 | ± | 13 |  | 67.2 | ± | 16.7 |  | 67.6 | ± | 14 |
| Distance traveled (m) | 1.3 | ± | 0.15 |  | 1.0 | ± | 0.12 |  | 1.0 | ± | 0.14 |  | 0.7 | ± | 0.1 |
| Results are expressed as means ±S.E.M. | | | | | | | | | | | | | | | |

**Table S8 Statistical analysis of the nest-seeking test parameters.**

| Call characteristic | Source of variation | F [DFn, DFd] / H [DFn, N] | p Value |
| --- | --- | --- | --- |
| Latency to the first nest zone entries | Treatment | H [1, N= 65] = 0.4 | 0.5277 |
|  | Sex | H [1, N= 65] = 0.19 | 0.6624 |
|  |  |  |  |
| Number of entries to nest zone | Treatment | H [1, N= 65] = 0.58 | 0.4477 |
|  | Sex | H [1, N= 65] = 0.52 | 0.4714 |
|  |  |  |  |
| Time spent in the nest zone | Treatment | H [1, N= 65] = 0.39 | 0.5323 |
|  | Sex | H [1, N= 65] = 0.04 | 0.8403 |
|  |  |  |  |
| Distance traveled | Treatment | F [1, 61] = 2.24 | 0.1393 |
|  | Sex | F [1, 61] = 3.54 | 0.0648 |
|  | Treatment × Sex | F [1, 61] = 0 | 0.964 |
|  |  |  |  |

**References:**

Scattoni, M. L., Puopolo, M., Calamandrei, G., & Ricceri, L. (2005). Basal forebrain cholinergic lesions in 7-day-old rats alter ultrasound vocalisations and homing behaviour. *Behavioural Brain Research*, *161*(1), 169–172. https://doi.org/10.1016/j.bbr.2005.01.011

Tonkiss, J., Harrison, R. H., & Galler, J. R. (1996). Differential Effects of Prenatal Protein Malnutrition and Prenatal Cocaine on a Test of Homing Behavior in Rat Pups. *Physiology & Behavior*, *60*(3), 1013–1018.
